# Design, Dynamic Modeling, and Motion Analysis of a Frog-Inspired Hybrid-Driven Amphibious Robot

**DOI:** 10.3390/s26133995

**Published:** 2026-06-24

**Authors:** Yitao Pan, Libing Hu, Yongsheng Ou, Jizhuang Fan

**Affiliations:** 1 School of Control Science and Engineering, Dalian University of Technology, Linggong Road 2, Dalian 116024, China; panyitao@dlut.edu.cn (Y.P.); hulibing@mail.dlut.edu.cn (L.H.); 2 State Key Laboratory of Robotics and System, Harbin Institute of Technology (HIT), Harbin 150001, China; techhit@126.com

**Keywords:** frog-inspired amphibious robot, jumping locomotion, swimming locomotion, hybrid drive

## Abstract

**Highlights:**

**What are the main findings?**

- This study proposes a novel frog-inspired hybrid-driven amphibious robot integrating combustion-driven jumping and cable-driven swimming mechanisms, enabling amphibious locomotion without complex structural reconfiguration.

**What are the implication of the main finding?**

- This scheme addresses the core pain point of traditional amphibious robots that rely on complex structural mode switching, not only providing a new design paradigm for small bionic amphibious robots, but also offering a reference benchmark for the subsequent research and development of frog-inspired amphibious robots.

**Abstract:**

To improve the amphibious locomotion capability of robots in aquatic and terrestrial environments, this paper proposes a novel frog-inspired hybrid-driven amphibious robot inspired by the amphibious locomotion characteristics of frogs. Unlike existing frog-inspired robots limited to single-mode jumping or swimming, this robot adopts an innovative hybrid actuation mechanism to simultaneously achieve frog-like swimming and jumping capabilities. On land, it uses a combustion-driven hindlimb propulsion mechanism paired with a linkage-based forelimb posture adjustment mechanism to realize frog-like jumping; in water, it employs a cable-driven linked hindlimb mechanism combined with a controllable soft extension-driven webbed foot to accomplish frog-like swimming. Furthermore, the instantaneous combustion thrust during frog-like jumping and the hydrodynamic thrust during swimming are calculated. The mapping relationships between the take-off attitude angle, hydrogen–oxygen mixture charge, and jumping performance, as well as the motion pattern between hindlimb motion parameters and swimming thrust, are derived. Finally, experimental results demonstrate that the robot achieves a swimming speed of 79 mm/s, a jumping height of 560 mm, and a jumping distance of 1200 mm, while being capable of performing continuous amphibious locomotion.

## 1. Introduction

In recent years, with the rapid development of science and technology, significant research breakthroughs have been made in various fields, including sensor technology [1], novel materials [2,3,4], 3D printing [5,6], and artificial intelligence. This advancement has effectively accelerated the development of robotics technology, enhancing its integrated operational capabilities and enabling robots to undertake high-risk or complex tasks that are difficult for humans to perform. However, the application scenarios of robots have gradually expanded from the initial unit-based manufacturing environments to various complex domains, including domestic services, battlefield operations, and extreme high-radiation environments in the nuclear industry [7,8]. Therefore, special tasks such as geological disaster rescue, shallow riverbed exploration, and amphibious battlefield reconnaissance require robots to complete missions successfully across diverse environments. With changes in environmental conditions, the locomotion modes of robots must also adapt accordingly. Generally, robots with a single locomotion mode are limited to operation in specific environments. For example, aerial robots [9] are capable of flying in the air but lack ground locomotion mechanisms, making them unsuitable for terrestrial movement. Ground mobile robots [10] exhibit effective mobility on land but are unable to operate underwater due to the absence of underwater propulsion systems. Similarly, underwater robots [11] maneuver freely in aquatic environments but cannot operate on land or in the air due to the absence of corresponding mobility mechanisms. In contrast to the aforementioned single-mode mobile robots, multi-modal mobile robots are designed with specialized structures that enable them to perform tasks across multiple environments. As a result, this type of robot [12,13,14,15,16,17] has garnered increasing attention and interest from researchers. Among them, amphibious mobile robots have shown great potential for application in both aquatic and terrestrial environments [18,19]. They are widely used in fields such as scientific exploration, marine ecological monitoring, and underwater resource exploration and also play a crucial role in tasks like natural disaster rescue, military reconnaissance, and coastal patrols [20,21,22,23,24]. Therefore, addressing the structural design, kinematic modeling, and motion planning of amphibious robots to meet the demands of both aquatic and terrestrial operations is of significant practical importance [25,26].

However, most existing amphibious robots rely on traditional locomotion systems (e.g., wheeled, tracked, legged, or hybrid configurations) to achieve cross-medium locomotion. In 2024, Lu et al. [27] from Huazhong University of Science and Technology proposed a wheel–propeller hybrid amphibious robot equipped with variable-pitch propellers. By adjusting the pitch angle of the propeller blades, the robot is capable of fast and stable locomotion on flat terrain, obstacle traversal, and omnidirectional movement on water surfaces. In 2025, Sato [28] from Okayama University of Science proposed a multi-joint propeller-based amphibious robot. By altering the joint orientation of the leg mechanism with a planar hinge, the robot achieves stable locomotion on both land and water surfaces. In 2023, Xu et al. [29] from Hohai University proposed a screw-propelled amphibious robot. By optimizing and designing the height and pitch of the screw blades, the robot achieves improved adaptability in semi-fluid amphibious environments, such as marshes and mudflats. In 2025, Hu [30] from Anhui Jianzhu University proposed a hybrid wheel–paddle-leg integrated amphibious robot. The robot adopts a composite wheel based on a planetary gear mechanism, which allows the circular wheel to morph into a paddle-leg configuration and enables multiple locomotion modes. This design allows the amphibious robot to operate effectively across diverse task environments. In summary, wheeled mechanisms feature simple structures, high propulsion efficiency, and ease of control, but exhibit limited environmental adaptability. Tracked and legged mechanisms offer strong obstacle-surmounting capabilities, yet are heavier, structurally more complex, and less maneuverable. Hybrid locomotion systems demonstrate better adaptability to complex environments; however, their mechanical structures and control systems are relatively complex to design. Overall, conventional amphibious robots are mature, reliable, and efficient, but they are significantly constrained by terrain conditions, which reduces their effectiveness in unfamiliar or complex operational environments.

Compared with traditional drive-type amphibious robots, biomimetic amphibious robots draw inspiration from the high flexibility [31] and strong adaptability [32,33] of biological organisms in unstructured dynamic environments, which endows them with richer and more flexible locomotion modes. This provides more design ideas for executing amphibious tasks in complex environments [34]. In 2022, a research team at Yale University [35] developed a biomimetic amphibious robot inspired by the locomotion characteristics of sea turtles. The robot uses variable-stiffness materials to fabricate flipper-like limbs. On land, the stiffness of the limb flippers is increased to make them rigid, enabling the robot to walk with a turtle-like limb oscillation. Once in the water, the stiffness of the flippers is reduced, transforming the limbs into fin-like appendages, allowing the robot to glide and swim in a turtle-like manner. In 2022, the University of Illinois in the United States [36], inspired by the locomotion mechanism of seals, developed a biomimetic amphibious robot. The robot consists of a wave-like deformable backbone mechanism and a spindle-shaped deformable support structure. By designing actuators based on shape memory alloys, the robot achieves rapid swimming in water and crawling motion on land. Similarly, in 2023, a research team from the National University of Defense Technology [37] developed a biomimetic undulating fin-propelled amphibious robot inspired by the undulating fin propulsion of aquatic animals. A series of undulating fin mechanisms were designed on both sides of the robot. Driven by composite wave motions, the robot is capable of fin-like swimming in water and crawling on land. In 2024, a research team from Nankai University [38], inspired by the undulatory motion of the flexible fins of stingrays, designed a novel traveling-wave-driven biomimetic stingray amphibious robot. The team developed a traveling-wave drive mechanism that converts the rotational motion of a motor into sinusoidal waves through a spiral rod passing through a series of aligned movable hinges. This design enhances both the robot’s mobility and its obstacle-crossing capability. Despite breakthroughs in mimicking the locomotion characteristics of natural organisms, the aforementioned bionic amphibious robot designs still exhibit several drawbacks. For example, these robots still rely on complex deformation structures and driving mechanisms, which result in more intricate control systems and higher susceptibility to malfunctions. Additionally, significant energy loss occurs during the deformation process, particularly during the transition between aquatic and terrestrial environments, which potentially reduces locomotion efficiency. Therefore, achieving simultaneous adaptability to both aquatic and terrestrial environments poses significant challenges [39,40] to the design and development of biomimetic amphibious robots, particularly in terms of their body structure, propulsion mechanisms, and motion control.

Compared to organisms such as turtles, seals, and rays, frogs, as typical amphibians, have a compact body and light weight, along with excellent swimming and jumping abilities both in water and on land. Inspired by the amphibious locomotion mechanism of frogs, researchers have developed biomimetic amphibious robots with explosive jumping capability and swimming, as well as exceptional amphibious agility. In 2007, Niiyama et al. [41] from the University of Tokyo developed a bipedal multi-joint biomimetic frog-inspired jumping robot, inspired by the skeletal features of frogs. The robot’s pneumatic muscles contract to drive the extension of its multi-jointed hind limbs, enabling it to achieve a vertical jump of up to 260 mm. In 2013, the Korea Advanced Institute of Science and Technology (KAIST) [42] developed a frog-inspired jumping robot, based on the structural principles of frog skeletal muscles. The robot utilizes a four-bar linkage mechanism to replicate the locomotion characteristics of the frog’s musculoskeletal system. By employing a torsional drive mechanism, it releases the elastic potential energy stored in the actuator, enabling the robot to achieve a jump height of 2.5 m. In 2021, Gao Feng [43] proposed a frog-inspired jumping robot based on explosive propulsion, targeting the high-energy, high-density explosive jumping mechanism of frogs. The robot was designed by simplifying the model of the frog’s hind and forelimb structures, using explosive propulsion as the actuator, achieving high explosive performance and continuous jumping motion. In 2022, Fan Jizhuang [44] from the Harbin Institute of Technology, inspired by the jumping motion of frogs, developed a novel bio-inspired frog jumping robot. The robot features a single-degree-of-freedom six-bar linkage hindlimb mechanism, which utilizes an energy storage spring and a triggering mechanism to achieve a maximum jumping height of 250 mm.

Similarly, researchers worldwide have drawn inspiration from the swimming characteristics of frogs and have developed various frog-inspired swimming robots based on different actuation mechanisms. In recent years, related studies have emerged. In 2015, Qiu Yulong [45] from the Harbin Institute of Technology, inspired by the biomechanical motion characteristics of frog hindlimbs, developed a frog-inspired swimming robot based on pneumatic muscles. The robot adopts a mechanism combining pneumatic muscle actuation, cable transmission, and spring-based resetting to achieve frog-like swimming. During the propulsion phase, the robot reaches an average velocity of up to 229 mm/s. In 2016, Kong Pengcheng [46] introduced improvements to the previously developed mechanism. By optimizing the design of a compact high-pressure air supply system, they enabled the robot to swim autonomously, achieving a propulsion speed of 0.5 m/s. In 2017, Zhang Wei [47] further enhanced the control system based on the aforementioned research. Inspired by the joint motion patterns of frogs, they designed a controller compatible with pneumatic muscle actuators to precisely control the joint movements, allowing the robot to reach a swimming speed of 790 mm/s in water. In 2017, researchers from the Department of Mechanical Engineering at the National University of Singapore [48] developed a frog-inspired swimming robot driven by dielectric elastomer actuators. The robot primarily features a hind-leg mechanism consisting of two joints and two webbed feet. Driven by the dielectric elastomer actuators, the webbed feet perform a complete movement cycle with a duration of 4 s, achieving an average swimming speed of 19 mm/s. In the same year, Gul [49] from Jeju National University in South Korea, inspired by the locomotion mechanism of frogs, developed a frog-like soft swimming robot based on shape memory alloys. The robot utilizes atomic layer deposition (ALD) technology to integrate flexible sensors into its limbs for real-time angle measurement. By activating the muscle actuators, the robot achieves coordinated movement between the torso and hind limbs, enabling it to perform basic frog-like swimming motions. In summary, although frog-inspired robots exhibit good locomotion performance in specific scenarios, they still face practical challenges such as low locomotion speed, poor environmental adaptability, and limited locomotion modes, which hinder their effective operation in amphibious environments. Moreover, most current researchers focus more on the bionic design of frog-like driving mechanisms, while lacking efficient trajectory planning methods tailored to the biological locomotion of frogs. For instance, Su et al. [50] proposed a trajectory planning method based on Dynamic Movement Primitives (DMPs) and Artificial Potential Field (APF) for tibial fracture reduction; Yuan [51] et al. developed a kinematic awareness-improved Hippopotamus Optimization Algorithm for path planning of mobile robots, among others. Although numerous motion planning methods [52,53] have been validated in the field of industrial robots, the application of these mature technologies in frog-inspired amphibious robots remains limited.

Based on the above analysis, existing amphibious robots typically adopt a mode-switching structure to achieve cross-medium locomotion. However, this approach results in complex mechanical structures, and such robots still face numerous challenges in waterproofing and structural maintenance. To address the aforementioned issues, this paper proposes a hybrid-driven frog-inspired amphibious robot inspired by the amphibious locomotion characteristics of frogs. The robot employs a hybrid drive system to achieve amphibious motion, including both frog-like swimming and jumping. On land, the robot adopts a combustion-driven hind limb propulsion mechanism and a linkage-based forelimb posture adjustment mechanism to achieve high-performance frog-like jumping. In water, a rope-driven linked rear limb mechanism performs extension and retraction movements, while a controllable soft-bodied extension-driven webbed foot mechanism is employed for oscillation and flexion, enabling efficient frog-like swimming. Compared with other amphibious robots, the proposed robot adopts a split design for its drive system, which significantly reduces the complexity of amphibious mode switching and improves its adaptability and controllability in both terrestrial and aquatic environments.

## 2. Structural Design of a Frog-Inspired Amphibious Robot

### 2.1. Structural Modeling Simplification of the Frog-like Amphibious Robot

**Figure 1:**
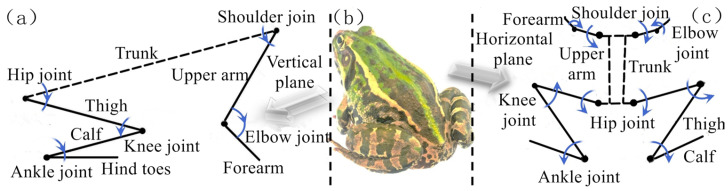
Simplified analysis of frog bone structure: (**a**) jumping mechanism; (**b**) real frog; (**c**) swimming mechanism.

Frogs live in complex amphibious environments, and with continuous evolution, their biomechanical mechanisms exhibit significant flexibility, resulting in complex movement patterns. Therefore, the robotic mechanism model design has yet to fully replicate the skeletal, muscular, and biomechanical motion characteristics of biological frogs. It is necessary to perform a reasonable abstraction and simplification of the frog’s skeletal structure, muscles, and movement characteristics. To enable the robotic model to replicate the multi-dimensional amphibious locomotion of frogs, the hip and shoulder joints are simplified as joints that move independently in two orthogonal planes (vertical and horizontal). As shown in Figure 1a, based on the analysis of the frog skeletal system for jumping motion, its hind limbs can be simplified into a vertical-planar linkage mechanism consisting of the hip joint, knee joint, ankle joint, tarsometatarsal joint, thigh, calf, and hind toes, where the hind toes are simplified from the phalanges and metatarsal bones. However, the forelimbs can be regarded as a planar linkage mechanism in the vertical plane, composed of the shoulder joint, elbow joint, upper arm, and forearm. Similarly, as shown in Figure 1c, based on the analysis of the frog skeletal system for swimming motion, its hind limbs can be simplified into a horizontal-plane linkage mechanism consisting of the hip joint, knee joint, ankle joint, thigh, calf, and webbed feet, where the webbed feet are simplified from the webbing between the tarsal bones, metatarsal bones, and phalanges. However, the forelimb can be regarded as a horizontal plane linkage mechanism, composed of the shoulder joint, elbow joint, upper arm, and forearm.

**Figure 2:**
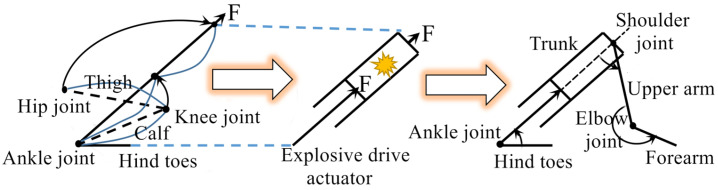
Abstract modeling of the robot’s jumping power mechanism.

Based on the above amphibious structural modeling of the frog, it can be concluded that when a frog jumps on land, the jumping height and distance depend on the explosive force of the hindlimbs. The hind limbs release energy through the rapid contraction of muscles, causing the thigh and calf in the hind limb mechanism to rapidly extend and align to a straight line, as shown in Figure 2. At this moment, the webbed feet of the hind limbs generate a strong reaction thrust against the ground. Therefore, to address the high-energy, high-density jumping drive characteristics, thigh, calf, hip joint, and knee joint of the frog’s hindlimb mechanism during jumping in the vertical plane are equivalent to the linear motion of an explosive drive actuator. This is combined with the ankle joint, hind toes, and forelimb mechanism, which are abstracted into a jumping mechanism model in the vertical plane. In this model, the ankle joint is referred to as the posture joint. Similarly, during the frog’s swimming motion, the swimming speed and distance are also determined by the explosive force of the hindlimb muscles. The hindlimb mechanism quickly extends the thigh and calf outward, causing the webbed feet to generate a reaction thrust against the water.

**Figure 3:**
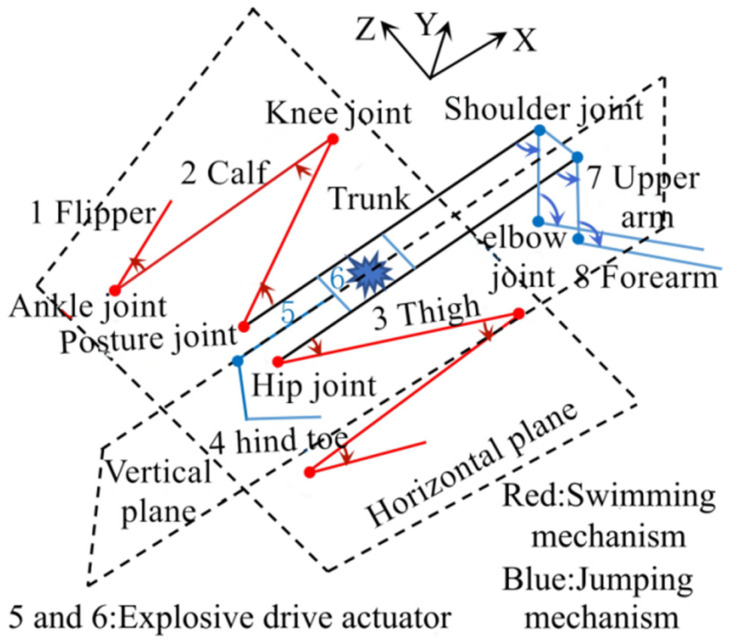
Mechanism model of hybrid drive amphibious robot inspired by frog.

Therefore, the swimming process differs from the jumping process in the following aspects: First, the area of the webbed feet needs to be adjusted during swimming, whereas no webbed foot expansion is required during terrestrial jumping. Second, the degrees of freedom of the forelimb joints and the locomotion of the elbow joint exert a negligible effect on overall acceleration, while during jumping, these joints can adjust the take-off posture angle and provide cushioning during landing impact. Based on the above analysis, the forelimb mechanism is omitted in the swimming motion modeling shown in Figure 1c, leaving only the hindlimb mechanism. In this mechanism, the hip and knee joints are driven by ropes, while the webbed feet are driven by a controllable extension-swing mechanism, abstracted as a swimming mechanism model in the horizontal plane. Finally, by integrating the jumping mechanism modeling from Figure 2, a frog-like hybrid drive amphibious robot structural model is designed, as shown in Figure 3.

### 2.2. Design of Frog-like Jumping Mechanism

**Figure 4:**
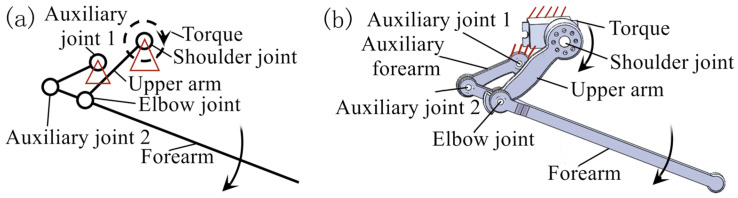
Design of linkage-based forelimb: (**a**) sketch of forelimb mechanism; (**b**) structure of forelimb mechanism.

Based on the aforementioned amphibian frog-like structural modeling, the robot’s jumping mechanism is inspired by the frog’s hindlimb explosive force and the agile adjustment of its forelimbs during jumping. This mechanism enables the robot to achieve jumping motion on land. It consists of a forelimb mechanism and an explosive actuator. Therefore, designing a mechanism that replicates the forelimb functions of frogs is critical for improving the jumping performance and stability of the proposed robot. As shown in Figure 4a,b), a multi-link mechanism is adopted as the robot’s forelimb structure to enable take-off posture adjustment and impact buffering during landing. From the perspective of the frog’s biological skeletal structure, the forelimb is small and structurally simple. The forelimb mechanism shown in Figure 4 has two degrees of freedom: the active degree of freedom at the shoulder joint and the passive degree of freedom at the elbow joint. Each forelimb mechanism is actuated by a small waterproof servo with a torque of 6 kg·cm. A linkage-driven mechanism is employed, in which the servo directly drives the upper arm to rotate about the shoulder joint, while the forearm is moved through mechanical linkage. Due to the large inertia during landing, it can cause impact damage to the upper arm, forearm, and joint axes. To address this issue, an auxiliary joint 1–2 and an auxiliary forearm are added at the intersection between the forearm and upper arm. This structure not only enhances the coupling motion of the shoulder joint through the auxiliary forearm and joint, but also increases the strength of the forelimb during the landing impact, allowing it to withstand greater forces and reduce damage to the forelimb caused by the interaction with the ground.

**Figure 5:**
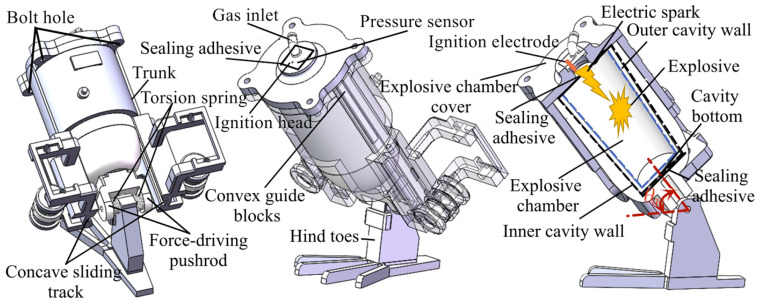
Design of soft-bodied cavity-type explosive actuator.

Secondly, frogs can generate powerful explosive force rapidly through their muscle tissue acting on the hindlimb skeleton, enabling them to achieve high-altitude and long-distance jumps. Based on this characteristic, a soft-material elastic and chemically explosive mechanism is employed to design a soft-bodied chamber-based explosive actuator, as shown in Figure 5. Its structure consists of an explosive module and a motion module. The explosive module is analogous to the muscle tissue responsible for force generation in the frog’s hind limbs. It consists of an ignition head, a soft-type explosive chamber, and a soft-type explosive chamber cover. The soft-type explosive chamber is the reaction chamber where the explosive reaction occurs. The upper surface of the chamber’s inlet is coated with sealing adhesive, which is then bonded to the lower surface of the explosive chamber cover. A hole is located at the center of the soft explosive chamber cover, which is connected to the lower surface. The ignition head is bonded to this hole using sealing adhesive. The ignition head is equipped with an ignition electrode, a gas inlet, and a pressure sensor. The motion module is analogous to the simplified linear motion output of the frog’s hind limb bones during a forceful jump. It consists of a force-driving pushrod and the hind toes. The outer sides of the force-driving pushrod are equipped with convex guide blocks, which allow the pushrod to move in a straight line within the body of trunk along a concave sliding track. Additionally, the force actuator is equipped with a cavity, whose shape and size are consistent with the soft explosive chamber. The soft explosive chamber is embedded within the cavity and is securely attached to the bottom of the force-driving pushrod’s inner cavity using a sealing adhesive. When the gas inside the explosive chamber detonates, the force-driving pushrod performs a rapid linear movement outward along the concave sliding groove relative to the robot trunk. Furthermore, torsion springs are embedded on both sides of the hinge between the lower end of the force-driving pushrod and hind toe. The torsion springs serve to quickly restore the hind toe to its original position after the robot completes a jump, utilizing the restoring force of the torsion springs, thus preparing the robot for the next jump.

In summary, under the control of the forelimb mechanism, the explosive actuator not only enables the robot trunk to achieve a predetermined take-off angle when the hind toes contact the ground, but also transmits the combustion force via the force transmission pushrod through the interaction between the hind toes and the ground, thereby generating a reaction thrust that propels the robot upward. Additionally, once the soft explosive chamber is filled with a hydrogen–oxygen mixture via the pneumatic control system, the main control board immediately activates the ignition system, causing the ignition electrodes to generate an electric spark that triggers combustion of the gas mixture within the chamber. This explosive event drives the explosive actuator to output thrust. Simultaneously, the forelimb mechanism adjusts the take-off angle between the robot’s trunk and hind toes. Under this configuration, the thrust generated by the explosive induces a propulsive force on the hind toes, enabling the robot to perform a frog-like jumping motion.

### 2.3. Frog-like Swimming Mechanism Design

**Figure 6:**
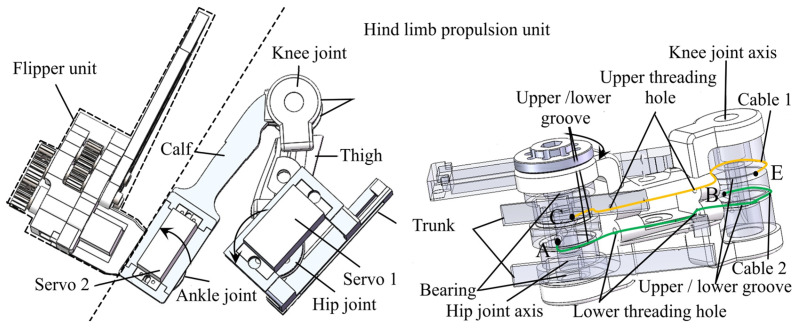
Design of the rope-driven linkage hind limb propulsion mechanism.

Based on the aforementioned structural modeling of frog amphibious locomotion, we design the swimming mechanism to replicate frog-like swimming by mimicking frog hindlimb extension/retraction and flipper expansion/contraction. The mechanism consists of a hind limb propulsion unit and a flipper unit, as shown in Figure 6. In this paper, by combining the flexibility of ropes with the rigidity of linkages, a rope-driven linked hindlimb propulsion mechanism is proposed. Since the initial state of the frog’s hind legs during swimming is always in a retracted position, the initial state of the rope-driven linked hindlimb propulsion mechanism is designed to be in a coiled position. The hindlimb propulsion mechanism consists of the thigh, calf, servo 1, servo 2, rope 1, and rope 2 and is connected to the trunk of the robot through the hip joint and knee joint. The upper end of the flipper mechanism is mounted onto the output shaft of servo 2 at the lower end of the calf, forming the ankle joint. During frog swimming, in addition to the movement of the flippers, the rotational degrees of freedom at the hip and knee joints of the hindlimbs must be controlled. To improve the efficiency of the drive source and reduce weight, the knee joint and hip joint are synchronized and controlled by a single servo 1 through a cable (1~2) linkage, enabling the synchronized outward/inward swinging degrees of freedom between the thigh and calf. In addition, the flipper mechanism is directly actuated by servo 2 to control its oscillatory motion. In the above design, to reduce weight while ensuring sufficient torque, servos (1~2) are respectively selected as small waterproof servos with torques of 6 kg·cm and 4.5 kg·cm, with weights of 25 g and 15 g, respectively. Additionally, to reduce frictional resistance during the relative motion between the hip joint and knee joint, rolling bearings are embedded in both axial ends of each joint.

**Figure 7:**
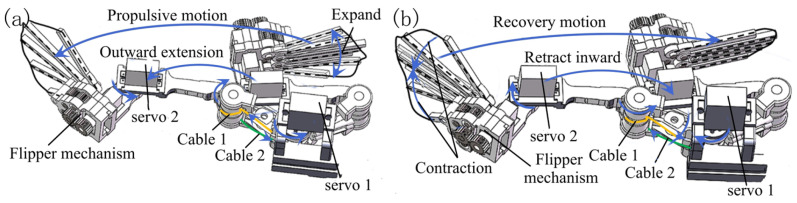
Motion state of the hind limb propulsion mechanism: (**a**) outward stretching exercise; (**b**) inward curling exercise.

When the thigh rotates counterclockwise around the hip joint axis via servo 1, cable 2 at point A will wind around the hip joint axis within the lower groove of the body trunk. As a result, the front end of the calf at point B will rotate clockwise around the knee joint axis to compensate for the length of the cable wound at the upper end. Meanwhile, cable 1 at point E will simultaneously wind around the knee joint axis within the calf’s front upper groove as the calf rotates clockwise around the knee joint axis. Therefore, the cable at the front end of the thigh at point C will rotate counterclockwise around the hip joint axis to compensate for the length of the cable wound at the upper end. Therefore, cables (1~2) exhibit a closed-loop coupling relationship characterized by equal length motion in opposite directions. Based on this coupling mechanism, as shown in Figure 7a, when servo 1 drives the thigh to rotate counterclockwise around the hip joint, it simultaneously drives the calf to rotate clockwise around the knee joint, resulting in an outward extension of the ankle joint. At this moment, the flipper mechanism expands the surface area of the flipper. As servo 2 subsequently swings counterclockwise around the ankle joint, the propulsion mechanism of the robot generates thrust for swimming motion. Conversely, as shown in Figure 7b, when servo 1 drives the thigh to rotate clockwise around the hip joint, the calf is simultaneously driven to rotate counterclockwise around the knee joint, causing the ankle joint to retract inward. At this stage, the flipper mechanism reduces the flipper surface area. As servo 2 then slowly swings clockwise around the ankle joint, the propulsion mechanism of the robot performs a recovery motion during the swimming cycle.

### 2.4. Body Design of the Frog-Inspired Amphibious Robot

**Figure 8:**
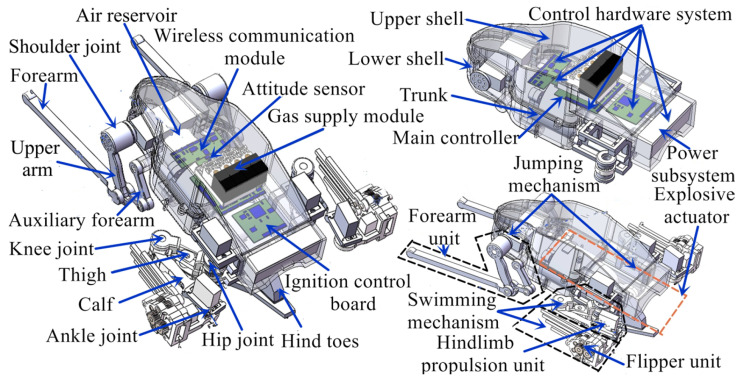
Overall model of hybrid drive amphibious robot inspired by frog.

Based on the design of the aforementioned amphibious locomotion mechanisms and integration of the control hardware system, the proposed frog-inspired hybrid-driven amphibious robot features integrated jumping and swimming capabilities. The robot adopts a hybrid actuation scheme combining cable-driven linkage actuation and combustion-driven propulsion, enabling long-distance untethered fully autonomous amphibious locomotion. The overall modeling structure is shown in Figure 8. The robot body shown in the Figure adopts an overall frog-like design and is mainly composed of the trunk mechanism, swimming mechanism, jumping mechanism, and control hardware system. The trunk mechanism mainly consists of an upper shell, a lower shell, and a central trunk body. A highly sealed cavity is achieved through a combination of static and dynamic sealing techniques; details regarding water sealing are provided in Files S3. It serves two main purposes: first, to increase buoyancy through increased water displacement; secondly, to create a sealed internal compartment functioning as an electronics bay, where the control hardware system is integrated. This configuration enables both the housing of the electronic control modules and structural connectivity with the lateral swimming and jumping mechanisms. Consequently, materials selected for the trunk structure and actuation units should exhibit high stiffness and hardness while maintaining a lightweight profile to optimize performance. The swimming mechanism adopts a cable-drive linkage system to mimic the extension and flexion of frog hindlimb muscles during swimming, enabling coordinated rotation of the hip and knee joints to achieve horizontal propulsion in a breaststroke-like motion. Meanwhile, a servo is integrated at the ankle joint to drive the flipper mechanism, allowing for both swinging motion and flipper opening and closing, thus enabling frog-like swimming motion underwater. The jumping power mechanism utilizes an explosive combustion-driven system to mimic the powerful burst generated by a frog’s hindlimb during jumping. In addition, the forelimbs assist with ground support and jumping angle adjustment during take-off and provide cushioning during landing, thereby enabling frog-like terrestrial jumping. The control hardware architecture comprises a gas supply module, ignition control board, main controller, pressure sensing unit, and power subsystem. Using a Wireless Communication Module, the host computer transmits control instructions to the main controller and receives real-time feedback, enabling accurate regulation of actuator dynamics. Each of the aforementioned modules operates independently while maintaining functional interdependence. Such a design ensures the robot’s capability to perform both swimming and jumping in amphibious environments, while also promoting a more compact, lightweight, and agile system architecture.

## 3. Hybrid Power Modeling of Frog-Inspired Amphibious Robots

### 3.1. Combustion-Explosion Dynamics Modeling for Robots

**Figure 9:**
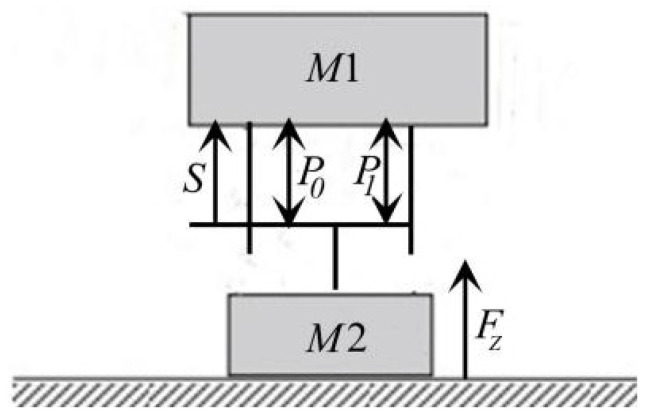
Schematic diagram of detonation dynamics model.

During the jumping process of the robot, explosive dynamics refers to the process in which the explosive actuator converts the chemical thermal energy generated by detonation into a reactive force output from the hind toe to the ground. Its simplified mathematical model is shown in Figure 9. The trunk, soft explosive chamber, chamber cover, and ignition head are simplified as the upper assembly with a total mass of M1. The force-exerting push rod and hind toes are simplified as the lower assembly with a total mass of M2. *S* denotes the motion stroke of the upper assembly relative to the lower assembly; *P*_0_ and *P*_1_ represent the initial pressure and the peak pressure during combustion within the soft combustion chamber, respectively.

According to the aforementioned combustion-based mathematical model, when the hydrogen–oxygen mixture undergoes detonation, the upper and lower assemblies generate a mutual motion S. By applying Newton’s second law, the dynamic equation of the upper assembly during motion S can be expressed as

(1) $$F_{M1}=\left(p_{1}-p_{0}\right)A_{q}-M1\cdot g-f$$

where *A_q_* denotes the deformable surface area of the soft actuator chamber (mm^2^); $F_{M1}$ represents the thrust force of the entire mechanism relative to the hind toe (N); and f is the friction force between the actuation push-rod and the torso body (N). During combustion-driven motion, the explosive force also actuates the driving pushrod, generating thrust at the hind toe. This results in a balanced state between the hind toe and the ground, and the corresponding dynamic equation is given as

(2) $$F_{M2}=\left(p_{1}-p_{0}\right)A_{q}-M2\cdot g-f$$

Due to the vertical contact between the hind toe and the ground, along with its rigidity, the explosive force generates a reaction thrust *F_Z_* from the ground. When this reaction thrust force *F_Z_* overcomes the total weight of both the upper and lower components, the system can rapidly detach from the ground, enabling a jump with a height H output. By combining Equations (1) and (2), the expression for the output force of the explosive actuator is given as

(3) $$F_{Z}=\left(p_{1}-p_{0}\right)A_{q}-\left(M1+M2\right)g-f$$

As shown in Equation (3), when the mixed gas inside the soft actuator chamber undergoes explosive combustion, the high-energy chemical reaction causes a pressure change within the chamber. This pressure difference P directly determines the output performance of the actuator. Since the combustion reaction occurs in a sealed soft actuator chamber, the first law of thermodynamics can be applied as follows:

(4) $${\overset{\cdot }{U}}_{\left(H_{2}+O_{2}\right)}={\overset{\cdot }{Q}}_{\left(H_{2}+O_{2}\right)}-{\overset{\cdot }{W}}_{\left(H_{2}+O_{2}\right)}$$

where *Q* represents the chemical energy released by the combustion of the hydrogen–oxygen mixture, U denotes the internal energy of the gas mixture, and *W* is the work done by the mixture on the external environment during the explosion process. Since the mass of the hydrogen–oxygen mixture filled in the chamber is relatively small, the combustion reaction within the soft chamber can be considered as a constant-volume, homogeneous, adiabatic, and complete combustion process. Based on the internal energy variation equation and expansion work formula of gas mixture during combustion explosion (see File S1 (Equations (S1) and (S2))), combined with Equation (4), the following expression can be derived:

(5) $$\overset{\cdot }{P}=-K_{\left(H_{2}+O_{2}\right)}\frac{P}{V}\overset{\cdot }{V}$$

Further integration of Equation (5) yields the following expression:

(6) $$P_{1}=P{\left(\frac{V_{1}}{V_{0}+A_{q}S}\right)}^{K_{\left(H_{2}+O_{2}\right)}}$$

where *V*_0_ is the initial volume of the hydrogen–oxygen mixture charged into the soft actuator chamber. Substituting Equation (6) into Equation (3), the blast-driven dynamic equation can be derived as follows:

(7) $$F_{Z}=\left(P{\left(\frac{V_{1}}{V_{0}+A_{q}S}\right)}^{K_{\left(H_{2}+O_{2}\right)}}-P_{0}\right)A_{q}-\left(M1+M2\right)g-f$$

### 3.2. Swimming Dynamics Modeling of the Robot

**Figure 10:**
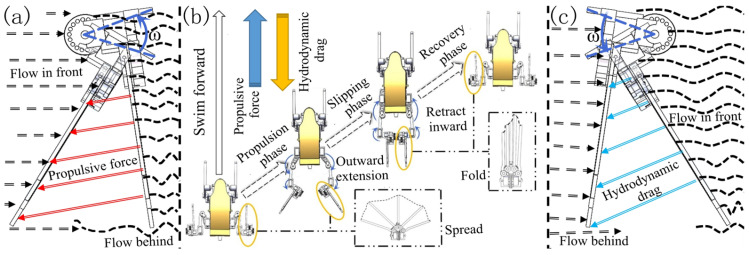
Changes in flippers subjected to water flow field: (**a**) propulsion stage; (**b**) motion state; (**c**) recovery stage.

As shown in Figure 10a, when the robot is in the propulsion phase, the flippers extend outward under the drive of the hind limb propulsion mechanism and spread to contact the water surface for swimming. At this point, the water flow speed behind the flippers increases relative to the water flow in front, generating a thrust in the direction of movement between the flippers and the water, referred to as the propulsive force. During the recovery phase, as shown in Figure 10c, the flippers retract inward under the actuation of the hind limb propulsion mechanism, and the flipper area is reduced while still interacting with the water surface. At this stage, the water flow velocity behind the flippers decreases relative to the front, resulting in a backward-directed force between the flippers and the water, opposite to the swimming direction. This force is referred to as hydrodynamic drag.

The robot’s side surface of the flippers has a very small width perpendicular to the horizontal plane of the flippers when moving through water, resulting in negligible lift on the side surface, which can be ignored. Considering the motion state generated by the flippers during the stroke, and combining hydrodynamic calculation methods, a hydrodynamic solution formula for the robot was established, as expressed below:

(8) $$F_{M}=C_{D}\rho_{S}\left(S\cdot \sin\left(\gamma \right)\right)U_{M}\left|U_{M}\right|$$

where $\rho_{S}$ denotes the density of the water flow, which is 1.0 × 10^3^ kg-m^3^; S denotes the effective contact area (mm^2^) between the flippers and the water during the stroke; γ denotes the effective angle between the contact area of the water flow and the flipper surface, ranging from 0° to 180°; $C_{D}$ denotes the drag coefficient of the flippers during the stroke; $U_{M}$ is the resultant velocity, combining the propulsion speed *V_o_* of the ankle joint in the base coordinate system and the linear velocity of the flipper at point *P*. The drag coefficient is determined by the Reynolds number *R_n_* for planar translation and rotation in the fluid flow; the calculation formula can be found in File S1 (Equation (S3)).

**Figure 11:**
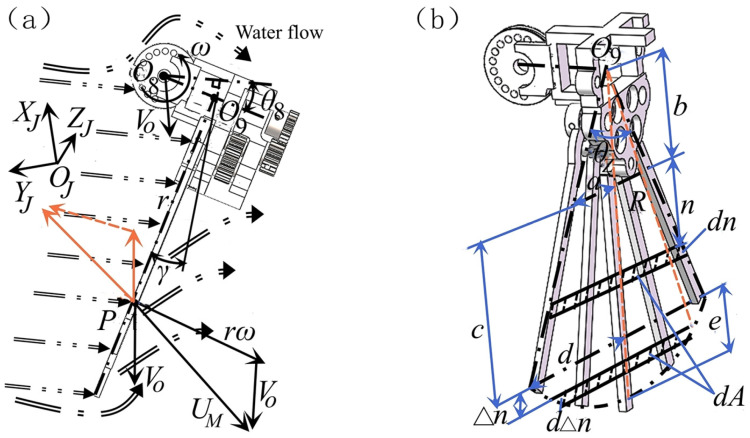
Force model of flipper motion in water: (**a**) force analysis of flipper motion; (**b**) basic parameters of flipper.

In Figure 11a, the distance between points *O*_8_ and *O*_9_ on the flipper mechanism is very short, so the angular velocity(*ω*) of point *O*_9_ is approximately equal to the angular velocity of point *O*_8_. In equation (8, *U_M_* is the resultant velocity of the ankle joint’s propulsion velocity *V_o_* in the base coordinate system and the linear velocity of the flipper at point *P*. The expression is as follows:

(9) $$U_{M}=V_{o}+\left(\omega \cdot r\right)$$

As shown in Figure 11b, the area of the flipper in the open position is approximately modeled as a sector with variable area. Using the blade element method, we consider all points on the flipper surface with the same velocity at point *P* as infinitesimal elements *dA*, with the rotation radius *r* as the variable. Then, by integrating Equation (8), the dynamic equation of the robot during swimming is derived. The expression is as follows:

(10) $$\begin{array}{cc} F_{M} & =0.5\int C_{D}\rho_{S}U_{M}\left|U_{M}\right|\sin\left(\gamma \right)dA\\ & =\;C_{D}\rho_{S}\left(\int_{0}^{c}\sin\left(\gamma \right)\left(\frac{d-a}{c}n+a\right){\left(V_{o}+\omega \cdot r\right)}^{2}dn\right)\\ & +\;C_{D}\rho_{S}\left(\int_{0}^{e}\sin\left(\gamma \right){\left(V_{o}+\omega \left(n+\Delta n\right)\right)}^{2}\sqrt{{\left(R\right)}^{2}-{\left(b+c+\Delta n\right)}^{2}}d\Delta n\right) \end{array}$$

where R represents the average length of the flipper’s toe bone, which is taken as 52 mm. The expressions for solving the basic parameters a, b, c, d, and e can be found in File S1 (Equation (S4)). By solving the above Equation (10), the thrust and hydrodynamic drag forces during the propulsion and recovery phases of the robot can be calculated with relatively low computational cost. Furthermore, by combining the robot’s swimming kinematic equations, it is possible to analytically determine the variation in hydrodynamic forces throughout the entire cycle, providing the foundation for subsequent trajectory planning and optimization.

## 4. Analysis of the Movement Capabilities in Frog-like Amphibious Robots

### 4.1. Frog-Style Jumping Motion

**Figure 12:**
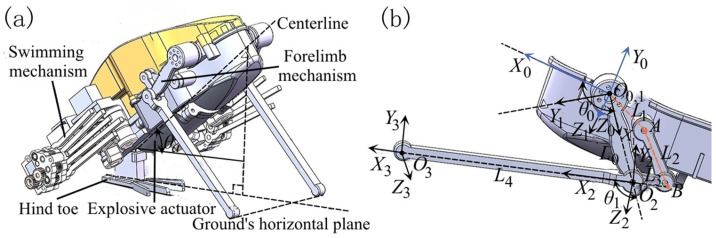
Robot jumping mode diagram: (**a**) robot posture angle; (**b**) coordinate system.

As shown in Figure 12a, the proposed jumping mechanism in this paper indicates that during the jumping process, the take-off posture is determined by the horizontal angle *θ_a_* between the force-driving pushrod in the explosive drive unit and the hind toe. The take-off posture angle is adjusted by the forelimb mechanism, which regulates the vertical distance between the robot’s centerline and the ground’s horizontal plane. The overall structure of the robot is symmetrically distributed. The coordinate system is established for the unilateral forelimb mechanism, as shown in Figure 12b.

According to the right-hand rule, coordinate systems *O*_0_*-X*_0_*Y*_0_*Y*_0_ and *O*_1_*-X*_1_*Y*_1_*Y*_1_ are established at the shoulder joint, the coordinate system *O*_2_*-X*_2_*Y*_2_*Y*_2_ is established at the elbow joint, and the coordinate system *O*_3_*-X*_3_*Y*_3_*Y*_3_ is established at the distal end of the forearm. Based on the link transformation matrix method, the D-H (Denavit–Hartenberg) parameter Table 1 for the left forelimb mechanism is obtained.

Based on the transformation matrix Tii−1 between adjacent joint generalized coordinate systems, the general form is

(11) Tii−1=cθi−sθi0ai−1sθicαi−1cθicαi−1−sαi−1−disαi−1sθisαi−1cθisαi−1cαi−1dicαi−10001

In the equation, *c* represents cos, *s* represents sin, and *i* denotes the *i*-th coordinate frame. Based on Equation (11) and the link parameters shown in Table 1, by multiplying the transformation matrices of each link, the transformation matrix of the forearm end-effector coordinate frame *O*_3_*-X*_3_*Y*_3_*Z*_3_ relative to the base coordinate frame *O*_0_*-X*_0_*Y*_0_*Z*_0_ can be obtained as follows:

(12) T30=T10θ0⋅T21θ1⋅T320

**Figure 13:**
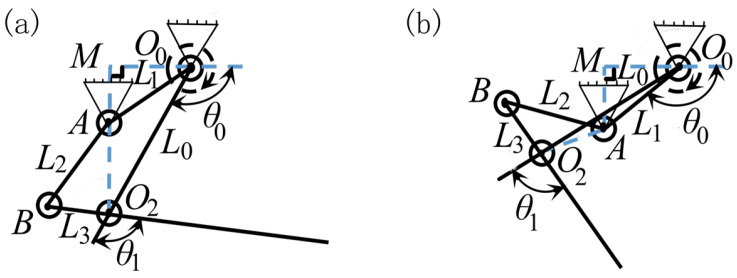
Schematic diagram of the forelimb mechanism motion: (**a**) does not exceed line segment *AO*_0_; (**b**) exceeds the line segment *AO*_0_.

In further rearranging Equation (12), the kinematic equation of the forearm end point in the left forelimb mechanism relative to the base coordinate frame of the shoulder joint, *O*_0_*-X*_0_*Y*_0_*Y*_0_, can be obtained as

(13) $$\left[\begin{array}{c} X_{3,0}\\ Y_{3,0}\\ Z_{3,0} \end{array}\right]=\left[\begin{array}{c} L_{4}\cdot \cos\left(\theta_{0}+\theta_{1}\right)+L_{0}\cdot \cos\left(\theta_{0}\right)\\ L_{4}\cdot \sin\left(\theta_{0}+\theta_{1}\right)+L_{0}\cdot \sin\left(\theta_{0}\right)\\ 0 \end{array}\right]$$

where (*X*_3,0_,*Y*_3,0_,*Z*_3,0_)*^T^* represents the position vector and *Y*_3,0_ is the motion equation of the forearm end point *O*_3_, which is the distance in the Y-direction perpendicular to the ground. As shown in Figure 13a,b, the forelimb mechanism adopts a multi-link design. Driven by the upper arm, the upper end point *B* of the forearm is constrained by the auxiliary forearm through a pulling mechanism, enabling the upper end of the forearm to swing forward and backward with the upper arm around the elbow joint *O*_2_. Therefore, a certain coupled motion relationship exists between the driving angles *θ*_1_ and *θ*_0_, and the decoupling computation is detailed in File S1 (Equations (S5)–(S11)).

Substituting the coupling motion relationship expression above into Formula (13) for calculation, the kinematic model of the forearm end-effector with respect to the driving angle is obtained as follows:

(14) $$\left[\begin{array}{c} X_{3,0}\\ Y_{3,0}\\ Z_{3,0} \end{array}\right]=\left[\begin{array}{c} L_{4}\cdot \cos\left(\theta_{0}\left(1+C\right)\right)+L_{0}\cdot \cos\left(\theta_{0}\right)\\ L_{4}\cdot \sin\left(\theta_{0}\left(1+C\right)\right)+L_{0}\cdot \sin\left(\theta_{0}\right)\\ 0 \end{array}\right]$$

**Figure 14:**
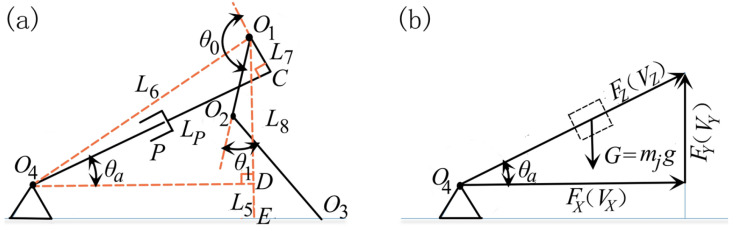
Schematic diagram of the robot’s jumping motion principle: (**a**) take-off posture angle; (**b**) jumping movement.

Based on the previously established model of the robot’s jumping actuation mechanism, the schematic diagram of the robot’s jumping motion was drawn, including the take-off posture angle and the jumping trajectory, as shown in Figure 14a,b.

According to the kinematic equations of the forearm end-effector derived above, the displacement *Y*_3,0_ of point *O*_3_ along the Y-axis determines the variation in the robot’s take-off posture angle. Given the right triangle △*O*_4_*CO*_1_ in Figure 14a, where the side lengths *L*_p_ and *L*_7_ are known, the length of side *L*_6_ can be derived using the Pythagorean theorem as follows:

(15) $$L_{6}=\sqrt{{\left(L_{p}\right)}^{2}+{\left(L_{7}\right)}^{2}}$$

Referring to the right triangle △O_4_CO_1_ in the figure, the included angle between the sides L6 and L7 can be determined.

(16) $$∠O_{1}O_{4}C=\arcsin\left(L_{7}/\sqrt{{\left(L_{p}\right)}^{2}+{\left(L_{7}\right)}^{2}}\right)$$

Similarly, based on the right triangle △O_4_DO_1_ in the figure, the included angle between sides L6 and L8 can be calculated.

(17) $$∠O_{1}O_{4}D=\arcsin\left(L_{8}/\sqrt{{\left(L_{p}\right)}^{2}+{\left(L_{7}\right)}^{2}}\right)$$

Given that the vertical motion equation of point O_1_ on the Y-axis is known, the length of *O*_1_*E* is *Y*_3−0_. Additionally, since the length of DE is *L*_5_, substituting into Equation (17) allows further calculation of

(18) $$∠O_{1}O_{4}D=\arcsin\left(\frac{Y_{3-0}-L_{5}}{\sqrt{{\left(L_{p}\right)}^{2}+{\left(L_{7}\right)}^{2}}}\right)$$

By combining Equations (17) and (16), the motion equation for the robot’s take-off posture angle is obtained, expressed as follows:

(19) $$\theta_{a}=\arcsin\left(\frac{Y_{3-0}-L_{5}}{\sqrt{{\left(L_{p}\right)}^{2}+{\left(L_{7}\right)}^{2}}}\right)-\arcsin\left(\frac{L_{7}}{\sqrt{{\left(L_{p}\right)}^{2}+{\left(L_{7}\right)}^{2}}}\right)$$

As shown in Figure 14b, the lengths of *L*_5_, *L*_7_, and *L_p_* are fixed values based on the prototype dimensions. Under the adjustment of the robot’s take-off posture angle, and in conjunction with the explosive dynamics Equation (7) in Section 3.1, a frog-like jumping motion on land is achieved. Given the driving force *F_Z_* from the explosive actuator on the robot’s rear toe, and applying the laws of energy and momentum conservation, the maximum initial velocity and total work of the robot during the explosive jump are determined:

(20) $$\{\begin{array}{c} V_{z}=\left(F_{Z}/m_{j}\right)\cdot T_{0}\\ W_{z}=\left(m_{j}\cdot {\left(V_{z}\right)}^{2}/2\right) \end{array}$$

where *m_j_* is the total mass of the robot (g) and *T*_0_ is the duration of the explosive actuation (s). During the robot’s jumping process, the explosive actuation occurs over an extremely short duration, approximately 0.2 ms. The jump is modeled as a projectile motion and is analyzed by decomposing it into vertical and horizontal components. Using Equations (19) and (20), the initial values of the horizontal and vertical velocities *V_X_* and *V_Y_* are derived:

(21) $$\{\begin{array}{c} V_{X}=\cos\left(\theta_{a}\right)\cdot V_{z}\\ V_{Y}=\sin\left(\theta_{a}\right)\cdot V_{z} \end{array}$$

The vertical velocity *V_Y_* undergoes uniform deceleration due to gravity until it reaches zero. During this time interval *T*_1_, the upward vertical displacement can be approximated as the jump height, where *T*_1_ = *V_Y_*/g. By combining the equations of velocity and displacement, the motion equation for the robot’s jump height is derived as follows:

(22) $$H=\left(2V_{Y}\sin\left(\theta_{a}\right)V_{z}-{\left(V_{Y}\right)}^{2}\right)/2g$$

When the robot reaches the maximum jump height *H*, it begins to fall to the ground under the influence of its own weight. The corresponding landing time can be calculated as follows:

(23) $$T_{2}=\left(\sin\left(\theta_{a}\right)V_{z}-\cos\left(\theta_{a}\right)V_{Y}\right)/g$$

From take-off to landing, the robot’s horizontal velocity component *V_X_* remains constant. The total time is *T*_3_ *= T*_1_ + *T*_2_. By combining the velocity and displacement equations, the robot’s jump distance motion equation is derived as follows:

(24) $$L_{Y}=\sin\left(2\theta_{a}\right)V_{z}^{2}/2g$$

### 4.2. Frog-Style Swimming Motion

**Figure 15:**
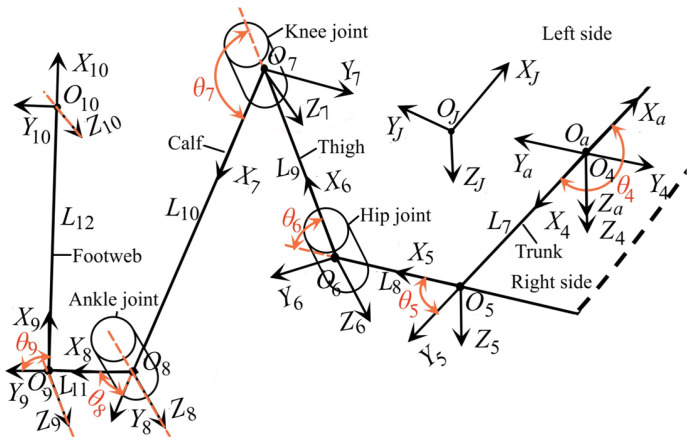
Kinematic model of robot swimming.

When the robot swims through the propulsion mechanism in water, the thigh, calf, and fin parts of the hind limb propulsion mechanism are simplified as a three-link hind limb mechanism model in the horizontal plane, as shown in Figure 15. The robot’s swimming mechanism is symmetrical, and the motion analysis is performed on its left side. The robot’s center of mass reference frame *O_a_-X_a_Y_a_Z_a_* moves in the world coordinate system *O_J_-X_J_Y_J_Z_J_*. Using the right-hand rule, the coordinate frames *O*_6_*-X*_6_*Y*_6_*Z*_6_, *O*_7_*-X*_7_*Y*_7_*Z*_7_, *O*_8_*-X*_8_*Y*_8_*Z*_8_, and O_10_*-X*_10_*Y*_10_*Z*_10_ are established at the hip joint, knee joint, ankle joint, and the end of the footweb in the hind limb mechanism, respectively. For computational convenience, several auxiliary coordinate frames have been added, namely *O*_4_*-X*_4_*Y*_4_*Z*_4_, *O*_5_*-X*_5_*Y*_5_*Z*_5_, and *O*_9_*-X*_9_*Y*_9_*Z*_9_.

**Figure 16:**
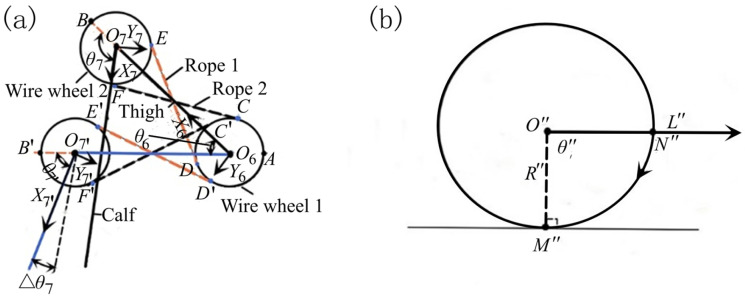
Movement relationship of the robot’s hind limb: (**a**) principle of rope-driven coupled motion; (**b**) principle of circular rolling.

Based on the joint coordinate frames shown in Figure 15, and using the D-H parameter method, the parameter table for the robot’s left hind limb mechanism (Table 2) is obtained, with the following data:

According to the transformation matrix between coordinate systems described in Equation (11), the parameters from Table 2 are substituted into the calculations. By multiplying the multiplication of the transformation matrices, the transformation matrix of the hind limb footweb end relative to the base coordinate frame *O_a_-X_a_Y_a_Z_a_* is obtained:

(25) T10a=T4a⋅T54⋅T65⋅T76⋅T87⋅T98⋅T109

where it represents the transformation matrix of the hind limb mechanism in the world coordinate system *O_J_-X_J_Y_J_Z_J_*, with the end coordinate frame *O*_10_*-X*_10_*Y*_10_*Z*_10_ relative to the base coordinate frame *O_a_-X_a_Y_a_Z_a_*. By simplifying and calculating Equation (25), the motion equation for the footweb end of the robot’s hind limb mechanism is obtained, with the following expression (***X_a_*_−10_**,***Y_a_*_−10_**,***Z_a_*_−10_**):

(26) T10a=Xa−10Ya−10Za−10=L10S67−L7+L9S6+L12C678+L11S678L12S678−L10C67−L9C6−L11C678−L80

where *C*_6_ represents cos(*θ*_6_); *C*_67_ represents cos(*θ*_6_ + *θ*_7_); *C*_678_ represents cos(*θ*_6_ + *θ*_7_ + *θ*_8_); *S*_67_ represents sin(*θ*_6_ + *θ*_7_); and *S*_678_ represents sin(*θ*_6_ + *θ*_7_ + *θ*_8_). As shown in Figure 16a,b, due to the rope-driven linkage motion mechanism employed in the robot’s hind limb mechanism, which includes the hip joint, knee joint, thigh, and lower leg, there exists a coupling relationship between the input of the hip joint angle and the output of the knee joint; the detailed calculations can be found in File S1 (Equations (S12)–(S17)).

Substituting the solution of the aforementioned coupled motion into Formula (26) with respect to T10a, the kinematic equation of the robot’s end-effector motion under the rear leg mechanism drive is further derived as follows:

(27) T10aθ6,θ8=Xa−10,Xa−10,Za−10T

The motion velocity of the ankle joint *O*_8_ in the rear leg mechanism is one of the factors influencing the robot’s propulsion and swimming dynamics in the subsequent hydrodynamic calculations. The propulsion motion equation of the ankle joint can be derived from Formula (27), and the expression is as follows:

(28) $$\{\begin{array}{c} X_{a-8}=L_{10}\sin\left(\theta_{6}\left(1+C_{7}\right)\right)-L_{7}+L_{9}\sin\left(\theta_{6}\right)\\ Y_{a-8}=L_{8}-L_{10}\cos\left(\theta_{6}\left(1+C_{7}\right)\right)-L_{9}\cos\left(\theta_{6}\right)\\ Z_{a-8}=0 \end{array}$$

where *C*_7_ represents the mapping kinematic relationship function of knee joint *θ*_7_, which is output by the hip joint linkage. By applying the time derivative method, the time derivative of the ankle joint motion Equation (28) is taken, resulting in the propulsion velocity motion equation of the robot’s driven flipper mechanism:

(29) $$\left[\begin{array}{c} {\dot{X}}_{a-8}\\ {\dot{Y}}_{a-8} \end{array}\right]=\left[\begin{array}{cc} L_{9}\cos\left(\theta_{6}\right)+L_{10}\cos\left(\theta_{6}+C_{7}\right) & L_{10}\cos\left(\theta_{6}+C_{7}\right)\\ L_{9}\sin\theta_{6}+L_{10}\sin\left(\theta_{6}+C_{7}\right) & L_{10}\sin\left(\theta_{6}+C_{7}\right) \end{array}\right]\left[\begin{array}{c} {\dot{\theta }}_{6}\\ {\dot{C}}_{7} \end{array}\right]$$

## 5. Experiment and Results

### 5.1. Prototype Hardware Platform Construction and Experimental Basic Configuration

**Figure 17:**
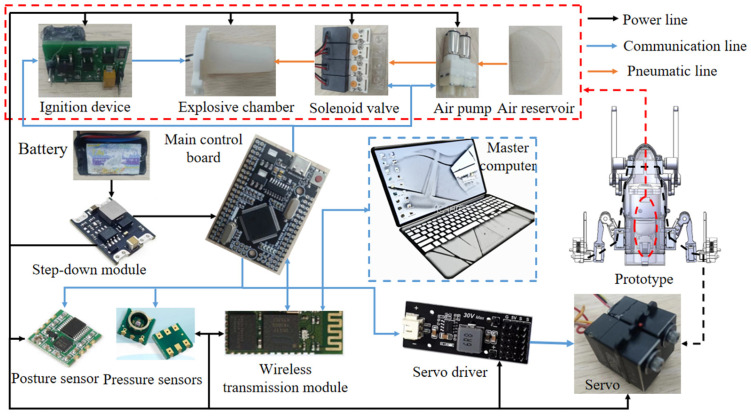
Hardware platform of the robot control system.

To enable the proposed robot to perform predefined locomotion tasks via the control program, we constructed the overall hardware architecture of the robot control system, as shown in Figure 17. Its hardware architecture includes the main control board, host computer, servos, ignition device, gas delivery system, attitude sensors, power supply, and step-down module, among others. The robot in this paper primarily integrates an independent ignition control hardware system, a pneumatic control hardware system, an electronic control hardware system, a wireless data transmission control hardware system, and a power supply control hardware system. The ignition control hardware system and the pneumatic control hardware system are responsible for controlling the explosive drive mechanism in the jumping propulsion system. They mainly consist of the gas delivery system and the ignition device. The electronic control hardware system is responsible for controlling the front limb mechanism, the hind limb propulsion mechanism in the swimming drive system, and the flipper mechanism. It primarily consists of servos and servo drivers. The wireless data transmission control hardware system manages data communication between the host computer and the main control board. It primarily consists of a wireless transmission module. The power supply control hardware system primarily provides power to the main control board, ignition control hardware system, pneumatic control hardware system, and electronic control hardware system. It mainly consists of a power supply and a voltage regulation module.

**Figure 18:**
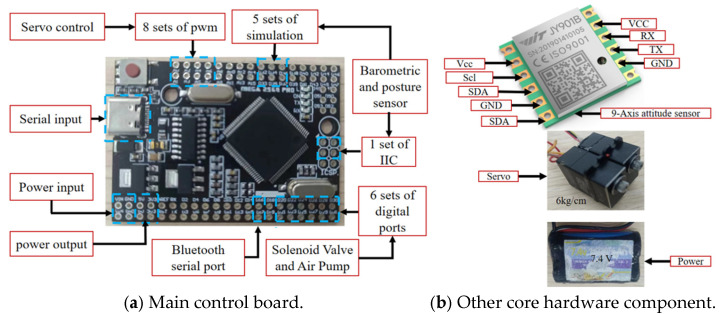
Major control hardware.

Based on the aforementioned control system, the designed robotic prototype is equipped with pneumatic hardware control, ignition hardware control, state measurement hardware control, and motion execution hardware control functionalities. In the pneumatic hardware control aspect, the system consists of a soft explosive drive chamber, four solenoid valves, two air pumps, and one hydrogen–oxygen storage tank. The soft explosive drive chamber requires the collaboration of two air pumps and four high-speed switching solenoid valves for inflating and deflating, thus necessitating six digital I/O ports for output. In the ignition hardware control aspect, one analog I/O port is required to trigger the ignition device to generate an arc. In terms of state measurement hardware, one nine-axis attitude sensor is needed, with a distance accuracy of 2 mm and an angular accuracy of ±1°, to detect the height, velocity, acceleration, and displacement during jumping and swimming motions. Additionally, four pressure sensors (GY-MS5837, with an accuracy of ±1 kPa) are needed to measure the pressure values in the gas storage tank and the soft explosive chamber. Therefore, a total of five analog I/O ports and one set of I^2^C interfaces are required. In the motion execution hardware control aspect, two servos controlled by Pulse Width Modulation (PWM) signals from the jumping power mechanism are responsible for the movement of the forelimb mechanism, while six servos controlled by PWM signals from the swimming power mechanism are used to control the movements of the hip joint, ankle joint, and the opening of the webbed feet. Therefore, eight analog I/O ports capable of outputting PWM signals are required, as shown in Figure 18b. In addition to the aforementioned battery, servos, and attitude sensor, please refer to File S2 for the hardware parameters, control principles, and configurations of the other relevant control system components.

In summary, conventional microcontroller control boards provide a limited number of I/O ports, whereas the proposed system requires the generation of PWM signals with adjustable frequency and high output accuracy, while also demanding substantial computational capability. Therefore, the Arduino MEGA2560(Arduino S.r.l., Monza, Italy), based on the ATmega2560 microcontroller (Microchip Technology Inc., Chandler, AZ, USA), was selected as the main control unit, as shown in Figure 18a. The main control board in the Figure is characterized by its compact size and integrated power output capability, enabling direct power supply to peripheral devices. The board provides 54 digital input/output (I/O) channels, including 14 PWM-capable channels, which offer sufficient control resources for various actuators, such as servo motors, solenoid valves, and pneumatic pumps. Additionally, it features 16 analog input/output channels for analog signal acquisition and PWM signal generation, thereby satisfying the interfacing requirements of pressure sensors, attitude measurement sensors, and ignition devices. The board also incorporates four TTL (Transistor Logic)-level UART (Universal Asynchronous Receiver/Transmitter) interfaces, supporting concurrent wireless data communication with multiple Bluetooth modules.

**Figure 19:**
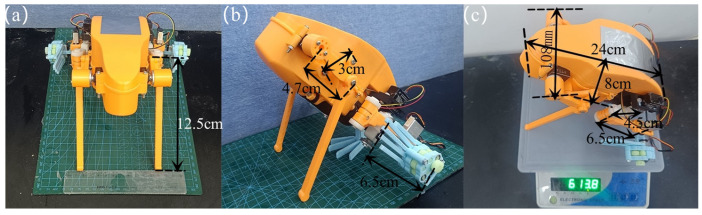
Overall physical prototype of the robot: (**a**) front view; (**b**) side view; (**c**) top view.

Combining the aforementioned hardware control system and the robot structure design discussed in Section 2, a prototype test platform has been built, as shown in Figure 19a–c. The actual dimensions of the robot’s front limbs are as follows: upper arm length 47 mm, lower arm length 125 mm, and auxiliary lower arm length 30 mm. The actual dimensions of the robot’s hind limbs are as follows: thigh length 45 mm, lower leg length 65 mm, and foot paddle length 65 mm. To provide sufficient drive space for the combustion actuator, two-thirds of the trunk length is allocated for the actuator space. The remaining portion, along with the space formed by the upper and lower housings, is used to house the electrical control hardware. Additionally, the head is designed to accommodate an 80 mL air storage tank. Therefore, the overall dimensions of the robot in its initial state are as follows: torso length 240 mm, shoulder width 108 mm, and torso height 95 mm, and the total mass is approximately 0.613 kg. Due to the requirement for the overall weight of the prototype to remain lightweight while maintaining a certain degree of rigidity, it is essential to ensure that it maintains a stable buoyant posture in water (for details, refer to File S4). Consequently, the shell material is selected to be nylon or resin with a density close to that of water; aside from color differences, all components are produced through a uniform 3D printing process. Furthermore, assuming the input quantity of each detonation is *V_L_* ml, the theoretical number of actuation cycles is calculated to be 80/*V_L_*, with a minimum of at least 1 cycle.

To further clarify the experimental implementation process and ensure the reproducibility of the research, the relevant experimental details, data acquisition procedures, and parameter settings are supplemented as follows. For the actuator timing, the complete jumping cycle sequence is gas filling (1.2 s), ignition delay (0.1 s), explosive actuation (~0.2 ms), and landing reset (~0.8 s); the complete swimming stroke cycle includes a propulsion phase (1.2 s), gliding phase (0.8 s), and recovery phase (1.0 s). The hydrogen–oxygen mixture (H_2_:O_2_ = 2:1) is filled into the combustion chamber through a pressure-feedback control system, and the volume is calibrated via the isothermal pressure–volume conversion law before experiments. For the data acquisition system, all experiments were recorded by a high-speed camera at 240 fps, with a standard scale backplane for spatial calibration (±2 mm accuracy). Onboard sensors included a 9-axis attitude sensor (WitMotion, Shenzhen, China) (100 Hz, ±1° angular accuracy) and four GY-MS5837 pressure sensors (Sanrui Technology Co., Ltd., Shenzhen, China) (200 Hz, ±1 kPa accuracy), all triggered synchronously by the Arduino MEGA2560 main control board. All sensors and the video capture system are calibrated with standard measuring tools before each batch of experiments. Each working condition was repeated five times independently, and three valid trials with the highest repeatability were selected according to preset criteria for final statistical analysis. Raw acceleration and pressure data were smoothed by a 4th-order Butterworth low-pass filter with a 10 Hz cutoff frequency in self-compiled MATLAB (25.2.0.2998904) programs, and all quantitative results are presented as standard deviation (SD). Swimming speed is defined as the average forward velocity of the robot’s center of mass in one complete stroke, extracted from high-speed video frame by frame. Jump height is the maximum vertical displacement of the center of mass from the take-off plane, and jump distance is the horizontal displacement from the take-off point to the first landing position. The comprehensive measurement uncertainty mainly comes from camera position error (±2 mm), attitude sensor angle error (±1°), and gas filling volume error (±0.5 mL), with calculated relative comprehensive uncertainty of ±4.2% for swimming speed, ±3.8% for jump height, and ±4.5% for jump distance. In terms of safety, the peak internal pressure of the soft combustion chamber under maximum filling volume (33 mL) is calculated based on the combustion dynamics model. Combined with the material pressure resistance test results in the design stage, the safety factor under maximum working condition is 2.3. Safety measures including over pressure relief valves, remote ignition, and isolation baffles are adopted in all experiments. Detailed valid trial screening criteria and the full data processing workflow are provided in File S5.

### 5.2. Prototype Frog-Style Swimming Test

**Figure 20:**
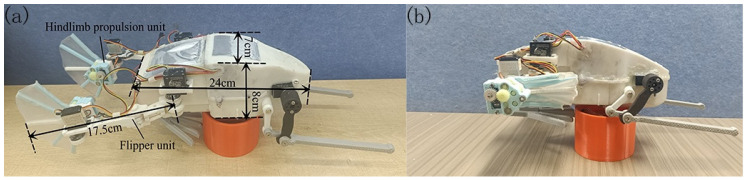
Layout of swimming power mechanism: (**a**) stretch state; (**b**) crouching state.

According to Section 3.2 and Section 4.2, the extension movement of the propulsion mechanism plays a decisive role in the robot’s swimming performance in water. The physical state of the propulsion mechanism on the robot prototype is shown in Figure 20a,b, with a fully extended length of 175 mm. The robot’s structure is more compact and lightweight.

**Figure 21:**
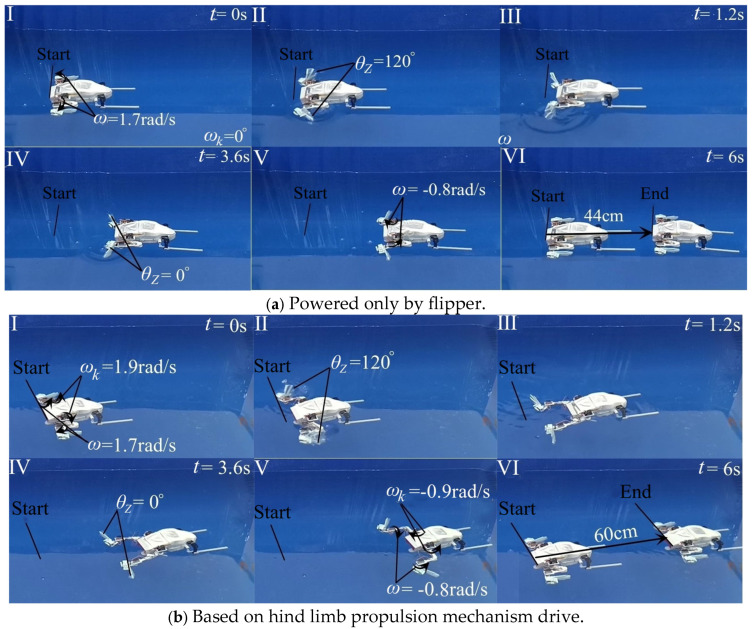
Robot swimming ability test experiment.

The frog’s swimming motion in water can be divided into three phases: propulsion, gliding, and recovery. Based on a host computer control program, the swimming actuation mechanism is regulated to enable the robot to perform frog-like swimming experiments in an aquatic environment. After processing the experimental video (See Video S1) and capturing screenshots, a sequence diagram of the robot’s swimming posture was obtained, as shown in Figure 21. In the figure, the ‘Start’ marker indicates the robot’s initial position, while the ‘End’ marker represents the robot’s final swimming position. In Figure 21a, the robot achieves straight-line swimming throughout the cycle by actuating only the lateral flippers with an angular velocity of 1.7 rad/s. The flipper opening angle is set at 120 degrees, resulting in a straight swimming distance equivalent to 1.8 times the body length of the prototype, approximately 440 mm, with an average speed of about 73 mm/s.

Figure 21b builds upon the control scheme in Figure 21a by additionally actuating the hip joints of the posterior limb propulsion mechanism at an angular velocity of 1.9 rad/s. This enhancement extends the straight-line swimming distance to 2.5 times the body length of the prototype, approximately 600 mm, with an average swimming speed of about 100 mm/s. By integrating the acceleration data obtained from the nine-axis orientation sensor during the experimental procedure (Data acquisition control program is provided in Data S1) and applying the physical relationship between force and acceleration, the propulsive force generated by the robot during the propulsion phase in water was experimentally estimated.

**Figure 22:**
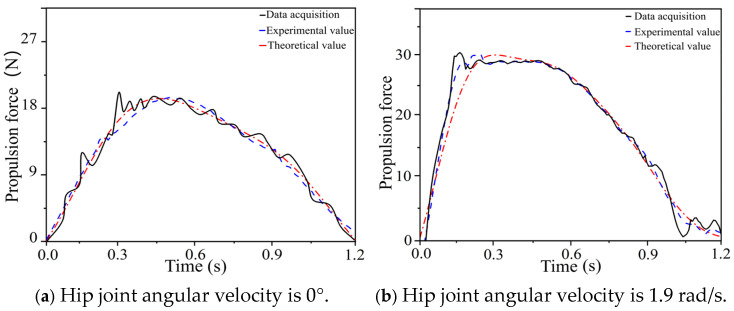
Propulsive force curve in propulsive phase.

At the same time, the theoretical propulsive force was calculated using the hydrodynamic Equation (10), and the results were plotted as shown in Figure 22a,b. As shown in Figure 22, the experimental results show that when the robot swims only with the flipper mechanism, the maximum thrust is approximately 22 N; with the addition of the hindlimb propulsion mechanism, the maximum swimming thrust increases to approximately 30 N. This indicates that the extension motion of the hindlimb mechanism increases the linear velocity of the flipper in water, and further enhances the propulsion force. According to recordings, the relative fluctuation of swimming thrust under steady operation is within 7%. Quantitative comparison shows that the average relative error between theoretical calculation and experimental results is 6.8%. The overall trend of experimental data is consistent with the theoretical model, which preliminarily demonstrates the feasibility of the swimming hydrodynamic model.

### 5.3. Prototype Frog-Style Jumping Test

**Figure 23:**
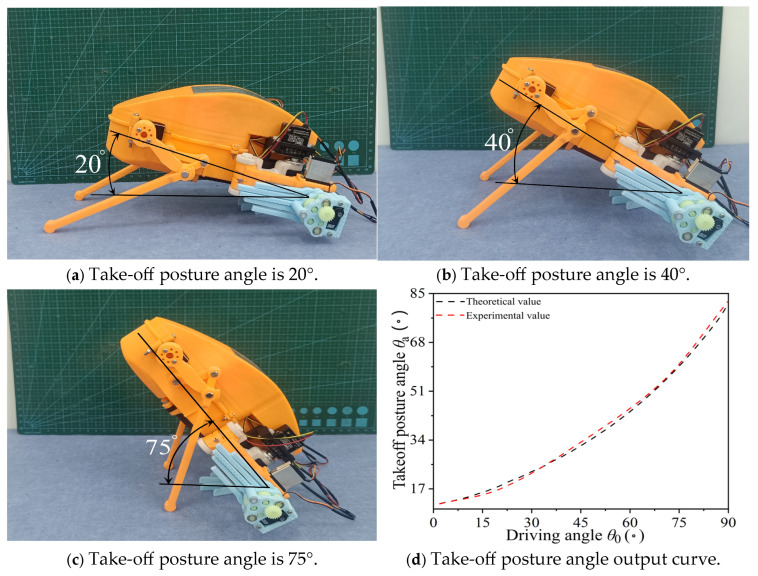
Change in the robot’s take-off posture angle adjustment.

Based on Equations (22) and (24) in Section 4.1, it can be concluded that the jump height and distance determine the robot’s jumping performance. The key parameters affecting this are the take-off posture angle *θ_a_* and the explosive force output *F_Z_* from the detonator. Therefore, the preferred approach in the experiment (see Video S2) is to drive the forelimb mechanism of the prototype, allowing the driving angle *Θ*_0_ to vary between 0° and 90°, which results in the change of the robot’s take-off posture angle *θ_a_* from 0° to 80°, as shown in Figure 23a–c. Meanwhile, the driving angle *θ*_0_ from Equation (19) is input with a specific value. After theoretical calculation, both the theoretical and experimental values (Data acquisition control program is provided in Data S2) are plotted together in Figure 23d. The error between the theoretical and experimental values in Figure 23d is minimal, and the trends align closely. This verifies the correctness of the theoretical derivation of the take-off posture angle and demonstrates the robot’s ability to adjust the take-off posture angle through its front limbs.

**Figure 24:**
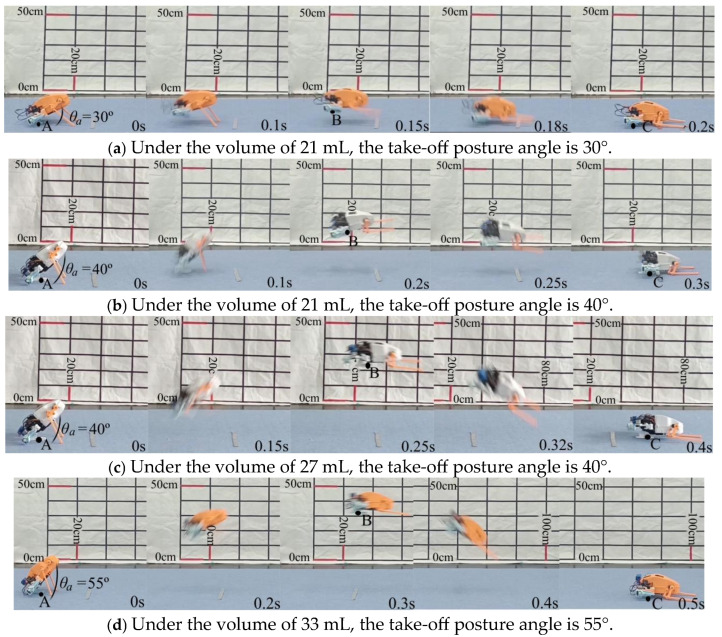
Robot jumping experiment.

Additionally, by programming the control of the forelimbs and the gas delivery system, the robot performed frog-like jumping experiments with varying amounts of hydrogen–oxygen mixed gas at different posture angles. In this experiment, the combustion chamber was a trapezoidal prism, and both the hydrogen–oxygen mixture ratio and the ratio of the chamber’s top and bottom surface areas were set to 2:1. By using a camera to record the entire experiment (See Video S3), key frames were extracted from the video to generate a sequence diagram of the robot’s frog-style jumping posture, as shown in Figure 24. In the motion sequence diagram, the corresponding transitional positions are annotated: point A represents the initial position, point B indicates the peak position, and point C denotes the final position.

**Figure 25:**
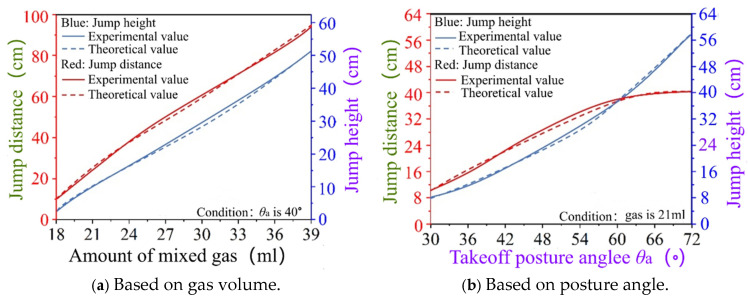
Robot jumping motion performance curve.

In Figure 24a, the robot’s take-off posture angle *θ_a_* is 30 degrees. During a single jump, it achieved a height of 50 mm and a distance of 100 mm. However, in Figure 24b, the robot adjusts the take-off posture angle *θ_a_* to 40 degrees. During a single jump, both the height and distance are significantly greater than those in Figure 24a, with a jump height of 100 mm and a jump distance of 230 mm. Similarly, compared to Figure 24b, with the take-off posture angle *θ_a_* unchanged in Figure 24c, the mixed gas volume is increased by 6 mL, resulting in a jump height of 230 mm and a jump distance of 460 mm. This outcome is significantly greater than the jump height and distance in Figure 24b. Compared to Figure 24c, the robot increases the take-off posture angle by 15 degrees in Figure 24d, the mixed gas volume is increased by 6 mL, resulting in a jump height of 330 mm and a jump distance of 820 mm. This outcome is significantly greater than the jump height and distance in Figure 24c. Therefore, it can be concluded that by altering the take-off posture angle and the mixed gas volume in the chamber, the robot can achieve different frog-like jump effects. Based on the above experiments, multiple experimental studies were conducted with take-off posture angle and the volume of mixed gas in the combustion chamber as input variables. Theoretical calculations were performed on Equations (22) and (24). The experimental data (Data acquisition control program is provided in Data S3) and theoretical values were compiled and analyzed, resulting in characteristic curves that reflect the impact of these factors on the robot’s jumping performance, as shown in Figure 25a,b.

From Figure 25a, it can be observed that when the robot’s take-off posture angle is fixed, as the amount of mixed gas injected into the detonation chamber of the explosive driver increases, the robot’s jumping height and distance will increase. From Figure 25b, it can be seen that when the amount of hydrogen–oxygen mixed gas injected is fixed, the robot’s jumping height increases as the take-off posture angle increases within the range of [30°, 72°]. The jumping distance increases as the take-off posture angle increases within the range of [30°, 60°], but decreases as the angle increases within the range of [60°, 72°]. This demonstrates that the robot’s frog-like jump height and distance increase approximately proportionally with the amount of mixed gas injected. The injected gas volume determines how high and far the robot can jump. The robot’s frog-like jump height increases approximately proportionally with the take-off angle, while the jump distance first increases approximately proportionally with the take-off angle and then decreases approximately inversely. The take-off angle determines the optimization of the robot’s efficiency in terms of height or distance during the jump. Additionally, according to records of valid trials under each working condition, the relative dispersion of jumping performance is within 8%. Quantitative comparison shows that the average relative error between theoretical calculation and experimental results is 5.2% for jump height and 7.1% for jump distance. The experimental results follow the theoretical trend well, which preliminarily verifies the rationality of the jumping dynamic model and take-off attitude adjustment mechanism.

### 5.4. Prototype Amphibious Frog-like Test

**Figure 26:**
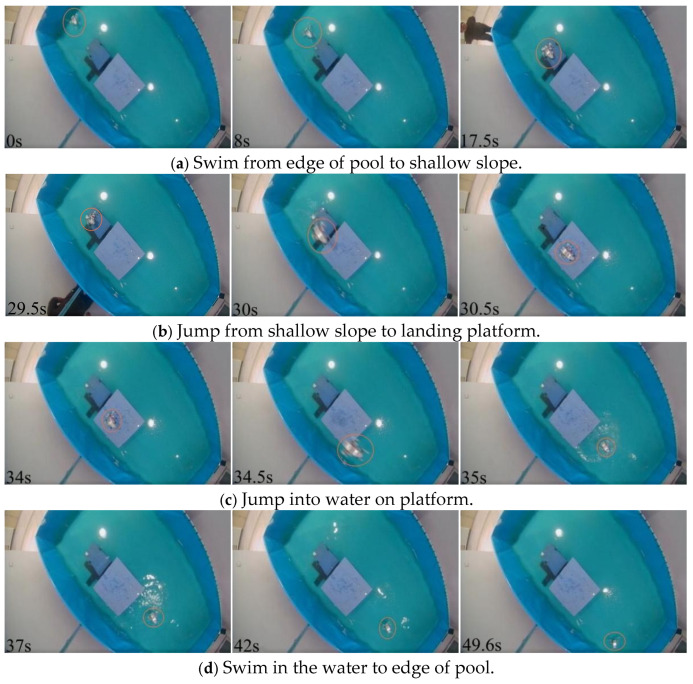
Robot amphibious movement experiment.

**Figure 27:**
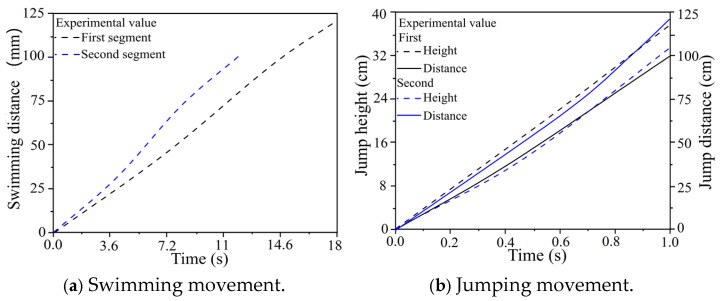
Changes in robot amphibious motion.

To further verify the amphibious mobility of the robot in a water–land alternating environment, a comprehensive experiment involving swimming and jumping was conducted on the robot. The experimental process consisted of swimming from the pool edge to a shallow slope, jumping from the shallow slope to a land platform, jumping from the land platform into the water, and then continuing to swim back to the pool edge. The pool was set with a length of 5000 mm, a width of 2000 mm, and a height of 800 mm. After recording with a camera, the experimental video results were processed into screenshots (see Video S4), resulting in a sequence of amphibious motion images of the robot, as shown in Figure 26a–d. Additionally, the data values obtained from the experiment (Data acquisition control program can be found in Data S4) are shown in Figure 27a,b. As shown in Figure 26a and Figure 27a, the robot completed three frog-like swimming strokes in the first segment within 17.5 s, successfully swimming from the pool edge to the shallow slope. The swimming distance was 1200 mm, with an average speed of 68 mm/s. Subsequently, the robot completed its first frog-like jump within 1 s, successfully leaping onto the land platform. The jump height and distance were 360 mm and 1000 mm, respectively, as shown in Figure 26b and Figure 27b.

Then, the robot completed its second frog-like jump within 1 s, leaping from the land platform into the water. The jump height and distance were 300 mm and 1200 mm, respectively, as shown in Figure 26c and Figure 27b. Finally, the robot completed three frog-like swimming strokes in the second segment within 12.6 s, successfully swimming from the water back to the pool edge. The swimming distance was 1000 mm, with an average speed of 79 mm/s, as shown in Figure 26d and Figure 27a. This validates the robot’s amphibious mobility, capable of both swimming in water and jumping on land.

Based on the aforementioned experimental research, Table 3 presents the specific performance parameters achieved by the robot during the experiments and compares these with existing typical frog-inspired robots. The results indicate that the prototype developed in this study achieves a swimming speed of 79 mm/s, a jumping height of 560 mm, and a jumping distance of 1200 mm at a weight of 613 g. Notably, the jumping height represents an improvement of 115% compared to the Mowgli robot (260 mm) and an enhancement of 124% relative to the robot developed by [44] (250 mm). Due to variations in the size of the prototype, its swimming speed is lower than that of specialized frog-like swimming robots. However, it simultaneously demonstrates the capability for both swimming and continuous jumping (≥2 times), showcasing superior amphibious locomotion abilities. Overall, the prototype proposed in this study achieves moderate levels for swimming speed, jumping height, and jumping distance, while exhibiting a lightweight design suitable for both aquatic and terrestrial environments, thus demonstrating excellent practical usability. Furthermore, this also validates the feasibility and correctness of the structural design, theoretical modeling, motion planning, and control systems of this type of frog-inspired hybrid-driven amphibious robot, laying a foundation for future research in bionic amphibious robotic locomotion.

## 6. Conclusions

To address the limitations of traditional amphibious robots, which rely on complex structural reconfiguration for locomotion mode switching, and the single locomotion mode of existing frog-inspired robots, this paper presents a novel frog-inspired hybrid-driven amphibious robot. The robot adopts a split hybrid-driven structure: on land, it achieves frog-like jumping through a combustion-driven hindlimb propulsion mechanism and a linkage-based forelimb posture adjustment mechanism; in water, it realizes frog-like swimming via a cable-driven linked hindlimb mechanism combined with a controllable soft extension-driven webbed foot, enabling amphibious mode switching without complex structural reconfiguration. On this basis, an instantaneous combustion dynamics model for the jumping process and a hydrodynamic model for the swimming process are established, and the mapping relationship between the take-off attitude angle, hydrogen–oxygen mixed gas charge, and jumping motion, as well as the influence law of hindlimb motion parameters on swimming thrust, are obtained. Finally, a lightweight prototype with a total mass of only 613 g is fabricated, and representative experiments on swimming and jumping locomotion are conducted. Experimental results show that the robot achieves a swimming speed of 79 mm/s, a jumping height of 560 mm, and a jumping distance of 1200 mm and can complete the continuous amphibious motion process of “aquatic swimming–terrestrial jumping–re-entry and swimming”. Within the test range, the experimental results are consistent with the theoretical trends, which preliminarily verifies the rationality of the proposed structural design, the feasibility of the dynamic models, and the practicability of the amphibious motion scheme. This work can provide a reference for the design of bionic amphibious robots.

## 7. Limitations and Future Work

While this study has validated the feasibility of the foundational design, dynamic modeling, and amphibious locomotion capabilities of the proposed frog-inspired hybrid-driven robot, several areas remain to be further explored in future research. For instance, repeated trials with statistical characterization will be performed to quantify the consistency of amphibious motion behaviors, complementing the representative performance data presented in this work. Further characterization of the explosive drive system will include measurements of the soft combustion chamber’s maximum operating pressure, safety margins and cyclic durability, as well as evaluations of heat dissipation and thermal stability during sustained jumping operations. We will also extend the current dynamic model to account for added mass and transient fluid drag effects during water–land transitions, verifying its validity under these complex boundary conditions. Additionally, this work only presents representative experimental results and lacks systematic statistical characterization based on a large number of repeated trials. The long-term cycle durability, thermal stability under continuous jumping, and comprehensive safety performance of the combustion-driven system have not been fully tested. In future research, we will optimize the durability of the soft combustion chamber, conduct complete repeated experiments with statistical analysis, and further improve the environmental adaptability of the robot in complex amphibious scenarios.

## Figures and Tables

**Table 1:** D-H parameter table of the robot’s left forelimb.

Joint Number (*i*)	*α_i−_*_1_ (°)	*a_i−_*_1_ (mm)	*d_i_* (mm)	*θ_i−_*_1_ (°)	Angle
1	0	0	0	*θ*_0_	80–130°
2	0	*L*_0_ = 47	0	*θ*_1_	−40–90°
3	0	*L*_4_ = 125	0	0	0

**Table 2:** D-H parameters of the left hindlimb mechanism of the robot.

Joint Number (*i)*	*α_i−_*_1_ (°)	*a_i−_*_1_ (mm)	*d_i_* (mm)	*θ_i_* (°)	Angle
4	0	0	0	*θ*_4_	−180°
5	0	*L*_7_ = 63	0	*θ*_5_	−90°
6	0	*L*_8_ = 42	0	*θ*_6_	−50°~70°
7	0	*L*_9_ = 45	0	*θ*_7_	0°~150°
8	0	*L*_10_ = 65	0	*θ*_8_	−80°~80°
9	0	*L*_11_ = 21	0	*θ*_9_	−90°
10	0	*L*_12_ = 65	0	0	0°

**Table 3:** Verifying robot design specifications.

Name	Weight	Swimming Speed	Jumping Height	Jumping Distance
Parameter	613 g	79 mm/s	560 mm	1200 mm

## Data Availability

Data are contained within the article.

## Supplementary Materials

The following supporting information can be downloaded at: https://www.mdpi.com/article/10.3390/s26133995/s1, 1. Experiment videos S1–S4 correspond to Figure 21, Figure 23, Figure 24, and Figure 26, respectively. 2. Datas S1–S6 Data acquisition control program. 3. Files S1 Detailed Derivation of Model Formulas. 4. Files S2 Main Control Hardware System. 5. Files S3 Design of the robot’s waterproof seal. 6. Files S4 Overall Stability of the Robot in Water. 7. Files S5 Detailed Data Acquisition Procedures.
