# Supplementary material for: Design, Dynamic Modeling, and Motion Analysis of a Frog-Inspired Hybrid-Driven Amphibious Robot
Source: Sensors (Basel). 2026 Jun 24;26(13):3995. doi: 10.3390/s26133995 (PMC13364168; doi:10.3390/s26133995)
Supplement: Supplementary file 1 [file sensors-26-03995-s001.zip › Files S1 Detailed Derivation of Model Formulas.pdf]

Detailed Derivation of Model Formulas

3.1 Combustion-Explosion Dynamics Modeling for Robots

A mixture of hydrogen and oxygen at a specific ratio is introduced into the soft actuator chamber, where both gases are assumed to behave as ideal gases. The internal energy change of the gas mixture during the combustion process can be expressed as follows:

$$\dot{U}_{(H_2+O_2)} = \frac{\left(\dot{P}V + P\dot{V}\right)}{K_{(H_2+O_2)} - 1} \tag{S1}$$

Where V denotes the volume of the hydrogen-oxygen mixture injected into the soft actuator chamber (ml), and K denotes the adiabatic index of the mixed gas, which depends on the gas composition, temperature, and pressure. The calculation formula for K is given by C_p/C_V. In a normal temperature environment, when hydrogen and oxygen react completely to form H_2O, K is approximately 1.3. The soft chamber is made of flexible silicone material. During the combustion process of the hydrogen-oxygen mixture inside the chamber, work is done accompanied by expansion. The expression for the work done during expansion is as follows:

$$\dot{W}_{(H_2+O_2)} = P\dot{V} \tag{S2}$$

3.2 Swimming Dynamics Modeling of the Robot

where R_n can be expressed as follows in both the laminar and turbulent flow regimes:

$$R_n = \frac{\rho_S \cdot |C_D| \cdot D}{\zeta} \tag{S3}$$

Where D is the characteristic length along the direction of the water flow velocity, with the maximum width of the flipper taken as 140 mm; When the fluid velocity is less than 1 m/s, the dynamic viscosity (ζ) of water is 1.01×10^{-3}Pa·s. Substituting the relevant parameters into equation（S3）to calculate the Reynolds number, if its value is less than $2*10^5$, the drag coefficient is 1.

The expressions for solving the basic parameters a, b, c, d, and e are as follows:

$$\begin{cases} a = b\tan\left(\dfrac{\theta_z}{2}\right) & c = R\cos\left(\dfrac{\theta_z}{2}\right) - b \\ d = R\sin\left(\dfrac{\theta_z}{2}\right) & e = R - b - c \end{cases} \tag{S4}$$

4.1 Frog-Style Jumping Motion

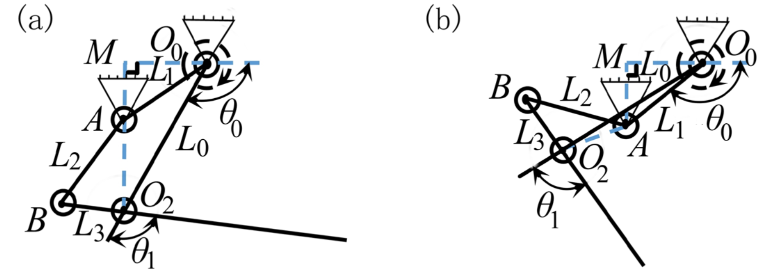

Figure S1. Schematic diagram of the forelimb mechanism motion:□ a□ Does not exceed line segment AO_0 ;□ b□ Exceeds the line segment AO_0

As shown in figure S1(a), when the upper arm rotates clockwise but does not exceed the position of segment AO_0, the three sides of right triangle $\triangle AMO_0$ are known, enabling the calculation of angle $\angle MO_0A$ via inverse kinematics. Given the driving angle of the upper arm θ_0, angle $\angle AO_0O_2$ can be further derived. The analytical expressions are as follows:

$$\angle AO_0O_2 = 180^\circ - \left(\theta_0 + \arccos\left(\frac{MO_0}{L_1}\right)\right) \tag{S5}$$

In the equation, the value of θ_0 is in the range [0□, 40□].Given triangle $\triangle AO_0O_2$ with two known sides and the included angle $\angle AO_0O_2$, the law of cosines is applied to derive the equation for the varying length of segment AO_2 during motion, as well as the angle $\angle AO_2O_0$. The expressions are as follows:

$$\begin{cases} AO_2 = \sqrt{(L_1)^2 + (L_0)^2 - 2L_1L_0\cos\angle AO_0O_2} \\ \angle AO_2O_0 = \arccos\left(\dfrac{(AO_2)^2 + (L_0)^2 - (L_1)^2}{2AO_2L_0}\right) \end{cases} \tag{S6}$$

Similarly, given triangle $\triangle ABO_2$ with all three sides known, the law of cosines in conjunction with equation (S6) is applied to derive the motion equation for the varying angle $\angle BO_2A$. The expression is as follows:

$$\angle BO_2A = \arccos\left(\frac{(AO_2)^2 + (L_3)^2 - (L_2)^2}{2AO_2L_3}\right) \tag{S7}$$

By summing the included angles derived from equations □ S6□ and □ S7□ , the mapping function of the elbow joint angle θ_1 with respect to the input angle θ_0 can be further obtained. The resulting expression is as follows:

$$\theta_1 = \angle AO_2O_0 + \angle BO_2A \qquad 0^\circ \le \theta_0 \le 40^\circ \tag{S8}$$

As shown in figure S1(b), when the upper arm rotates clockwise past the position of segment AO_0, the angle $\angle AO_0O_2$ can be determined based on the known angle $\angle MO_0A$ and the input angle θ_0 of the upper arm. The expression is given as follows:

$$\angle AO_0O_2 = \theta_0 - \left(180^\circ - \arccos\left(\frac{MO_0}{L_1}\right)\right) \tag{S9}$$

In the equation, the value of θ_0 is in the range [40□, 90□]. By substituting Equation □ S9□ into Equation □ S6□ , the motion length change equation for segment AO_2 and the angle $\angle AO_2O_0$ can be determined. Through the application of the motion variation equation of angle $\angle BO_2A$ from Equation□ S7□ and

performing a subtraction operation, the motion variation equation of the elbow joint angle θ_1 in relation to the input angle θ_0 can be obtained, as follows:

$$\theta_1 = \angle BO_2A - \angle AO_2O_0 \qquad 40^{\circ} \le \theta_0 \le 90^{\circ} \tag{S10}$$

By combining Equations □ S8□ and □ S10□ , the kinematic equation relating the driving angle θ_0 and the elbow joint angle θ_1 of the forelimb mechanism throughout the entire motion process can be derived as follows:

$$\theta_1 = \begin{cases} \angle BO_2A + \angle AO_2O_0 & 0^{\circ} \le \theta_0 \le 40^{\circ} \\ \angle BO_2A - \angle AO_2O_0 & 40^{\circ} \le \theta_0 \le 90^{\circ} \end{cases} \tag{S11}$$

It can be seen from Equation □ S11□ that L_0, L_1, L_2, L_3, and M_0 are predetermined design constants, with θ_0 being the sole variable parameter affecting the mechanism. Therefore, it can be further simplified as $\theta_1 = C{\cdot}\theta_0$, where C represents the functional relationship defined in Equation □ S11□ .

4.2 Frog-Style Swimming Motion

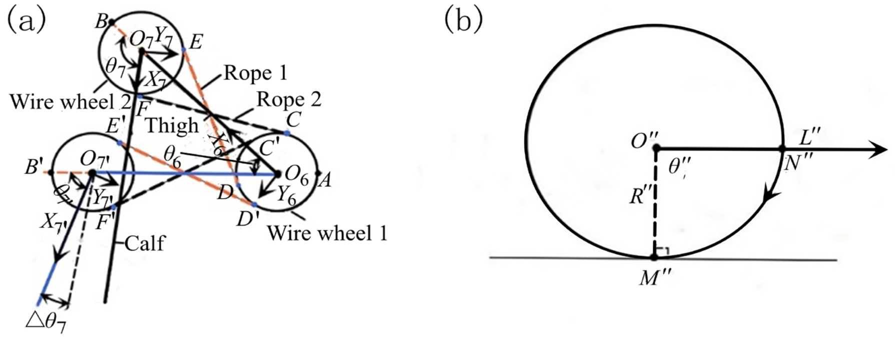

Figure S2. Movement relationship of the robot's hind limb:(a)Principle of rope-driven coupled motion;(b)Principle of circular rolling

Given that in figure S2 (b), the circle O'' rolls clockwise from point N'' to point M'' by an angle θ'', the arc length L'' of the forward movement of the center of circle O'' is obtained as follows:

$$L^{"} = 2R^{"}\pi\theta / 360^{\circ} \tag{S12}$$

As shown in figure S2 (a), when the thigh O_6O_7 rotates counterclockwise by an angle θ_6 around point O_6 as the center, the wire wheel 1 also rotates counterclockwise by an angle θ_6 around point O_6 with a radius R1. The value of ΔH is obtained according to the above formula □ S12□ □

$$\Delta H = 2\theta_6 R_1 \pi / 360^{\circ} \tag{S13}$$

Since the length of DB on rope 1 is shortened by ΔH, the endpoint B of the wire wheel 2 on the lower leg will be pulled by rope 1 to point B', and will rotate clockwise around point O_7 with a radius R_2 by an angle $\Delta\theta_7$. The rotation angle is calculated according to formula □ S13□ □

$$\Delta\theta_7 = \Delta H \cdot 360^{\circ} / \left(2\pi R_2\right) \tag{S14}$$

By substituting formula (S13) into formula (S14), the result is obtained as follows:

$$\Delta\theta_7 = \theta_6 R_1 / R_2 \tag{S15}$$

Since the initial angle of the lower leg mechanism is θ_7, after the angular change described by formula (S15), the resulting changed angle is obtained as follows:

$$\theta_{7'} = \theta_7 - \Delta\theta_7 \tag{S16}$$

By substituting formulab (S15) into formula(S16), the kinematic geometric relationship expression between the knee joint angle output and the hip joint angle input is further obtained as follows:

$$\theta_{7'} = \left[\theta_7 - \left[\frac{\theta_6 \cdot R_1}{R_2}\right]\right] \tag{S17}$$
