# Supplementary material for: Design, Dynamic Modeling, and Motion Analysis of a Frog-Inspired Hybrid-Driven Amphibious Robot
Source: Sensors (Basel). 2026 Jun 24;26(13):3995. doi: 10.3390/s26133995 (PMC13364168; doi:10.3390/s26133995)
Supplement: Supplementary file 1 [file sensors-26-03995-s001.zip › Files S2 Main Control Hardware System.pdf]

Main Control Hardware System

1. Pneumatic Control Hardware System

The hydrogen–oxygen-fueled combustion driver imposes higher performance requirements on the gas control and delivery system, thereby necessitating the development of a stable, reliable, miniaturized, and high-precision pneumatic control hardware system. However, existing gas delivery systems generally suffer from drawbacks such as large volume, excessive weight, and poor portability.As shown in figure S3, to overcome the aforementioned limitations, a pneumatic conveying control system was designed, consisting of two air pumps, four 2/2-way solenoid valves, three pressure sensors, and one valve guide.The gas transportation device comprises solenoid valves, pressure sensors, and a valve guide, with its assembly configuration presented in figure S3(a). As shown in figure S3(b), the valve guide functions as the central element of the gas-flow distribution network. Solenoid valves 1~4 are installed at locations a~d on the valve guide to control the opening and closing of ports A~D corresponding to the hydrogen chamber, oxygen chamber, intermediate chamber, and combustion chamber, respectively, thereby enabling selective routing, connection, and isolation of the associated gas flow channels.Pressure sensors are mounted beneath the valve guide to continuously monitor the pressure of the gas delivered to the intermediate chamber and the combustion chamber.As shown in figure S3(c), Pump 1 is responsible for extracting the hydrogen–oxygen gas mixture from the intermediate cavity and delivering it into the soft combustion chamber, whereas Pump 2 is used to evacuate the exhaust gases from the soft combustion chamber and discharge them into the atmosphere. Therefore, the pumps serve as the power source of the gas delivery system, providing the driving force required for gas transport.

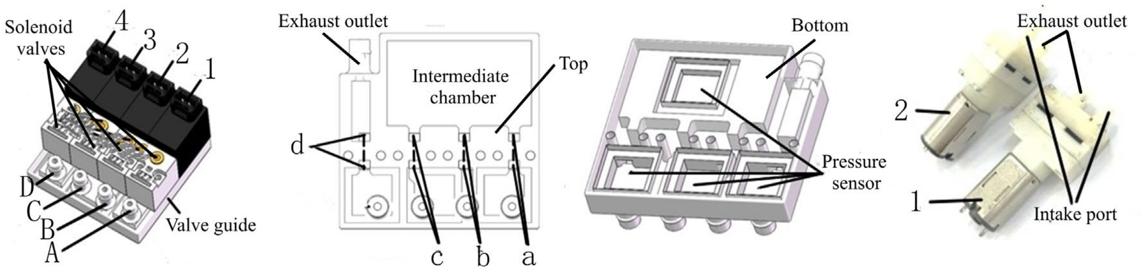

a）装配体　　b）阀导　　c）气泵

a) Assembly　　b) Valve guide　　c) Air pump

Figure S3 Components of pneumatic control conveyance system

As shown in figure S4, the procedure begins by activating solenoid valve 3 and starting air pump 2 to evacuate the air from the soft body combustion chamber. Subsequently, solenoid valve 4 is opened, and air pump 1 is activated to create a negative pressure in the intermediate chamber by drawing gas into the soft body combustion chamber. Next, solenoid valves 1 and 2 are engaged to allow hydrogen and oxygen from the storage bags to flow into the intermediate chamber in a predetermined volumetric ratio. Once the pressure in the intermediate chamber returns to the initial pressure value, solenoid valves 1 and 2 are promptly closed. Afterward, the process of opening solenoid valve 4 and activating air pump 1 is repeated to transfer the mixed gases from the intermediate chamber to the soft body combustion chamber. By controlling the number of cycles of the aforementioned process, precise regulation of the inflating volume can be achieved. Ultimately, the desired proportion and volume of the hydrogen-oxygen mixed gas can be safely and stably introduced into the soft body combustion chamber. The detailed control program for the inflating process during the experiment can be found in Data S5.

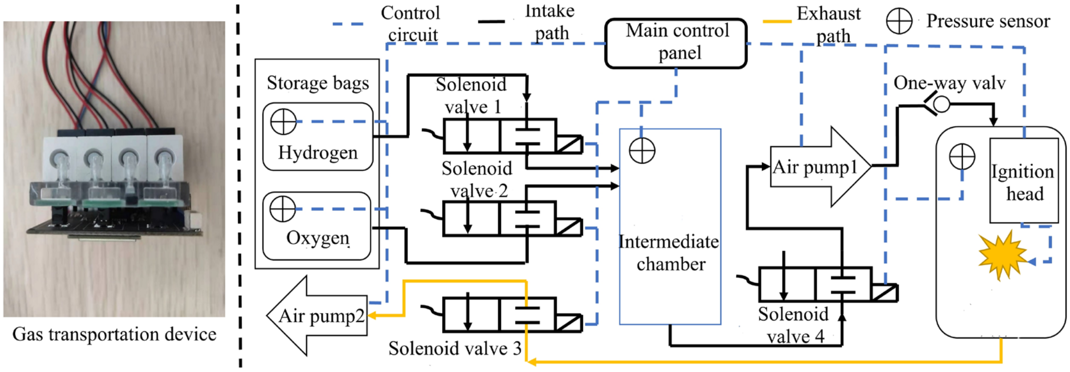

Figure S4 Pneumatic control conveyance working principle diagram

To ensure a dual safety measure and maintain isolation from the gas circuit, a one-way valve has been installed between air pump 1 and soft body combustion chamber. The role of the pressure sensor within the system is to provide real-time feedback on pressure variations in each section to the main control panel, thereby generating corresponding control commands to adjust the gas delivery, creating a closed-loop operation. To prevent any issues with the prototype during the experiment that could lead to a combustion reaction due to the mixed gases, an emergency safety stop procedure has been implemented in the program, as detailed in Data S6.

2. Ignition Control Hardware System

In order to achieve instantaneous high-temperature ignition of the gas reaction by breaking down the mixed explosive gas, the breakdown voltage is approximately 30 kV/cm. Given that the distance between the electrodes of the designed ignition head is 2 to 4 mm, it is necessary to instantaneously increase the voltage across the electrodes to over 10,000 volts to generate an electric spark. If the 7.4 V voltage is directly boosted to over 10,000 volts, a larger-sized step-up transformer would be required, which is not conducive to the miniaturization and weight reduction of the robot.As shown in figure S5, the ignition control system designed in this paper is capable of rapidly igniting the mixed gas while precisely controlling the timing of the explosion. Additionally, it features a compact structure, small size, and lightweight. The system consists of an ignition device and an ignition head, with the ignition device primarily composed of a 470 μF capacitor, an NPN transistor, an EL357 chip, a QAY212S chip, a 1:150 step-up transformer, and a 33 Ω, 1 W resistor, among other components.When the ignition device is powered with a voltage ranging from 3.3 V to 7.4 V, the current passes through the EL357 chip and NPN transistor, continuously charging the capacitor (470 μF, 16 V). The signal pin C1 of the QAY212S chip is connected to the IO pin of the main control board. When the C1 pin receives a low-level control signal from the IO pin, it triggers an immediate discharge of the capacitor, which is then stepped up rapidly through the transformer, elevating the output voltage to approximately 10-20 kV. At this moment, the output is connected to the electrodes on the ignition head, which are made of metal header pins. Under the high voltage of 10-20 kV, the gas medium breaks down, producing a high-temperature electric spark capable of igniting a hydrogen-oxygen mixture. When the C1 pin receives a high-level control signal from the IO pin, the ignition device outputs 0 V, and the ignition on the electrodes halts immediately. Thus, by controlling the duration of the low-level signal at the IO pin of the main control board, the ignition timing between the electrodes can be precisely managed.

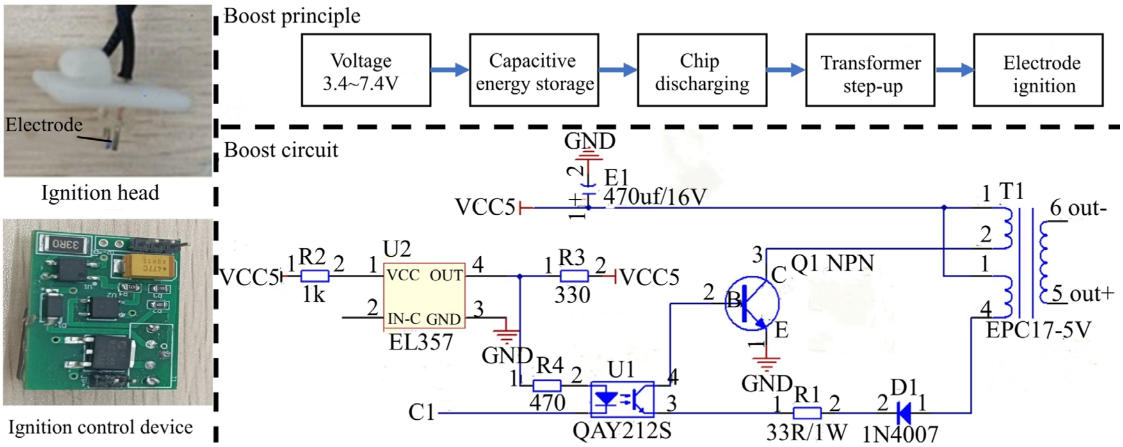

Figure S5 Ignition control system design
