# Supplementary material for: Design, Dynamic Modeling, and Motion Analysis of a Frog-Inspired Hybrid-Driven Amphibious Robot
Source: Sensors (Basel). 2026 Jun 24;26(13):3995. doi: 10.3390/s26133995 (PMC13364168; doi:10.3390/s26133995)
Supplement: Supplementary file 1 [file sensors-26-03995-s001.zip › Files S5 Detailed Data Acquisition Procedures.pdf]

Detailed Data Acquisition Procedures

1. Test Equipment and Parameter Settings

The experimental data acquisition system consists of three modules: motion capture module, onboard sensing module, and calibration auxiliary module.

Motion capture module: A high-speed camera with a resolution of 1920×1080 was used to record the full locomotion process of the robot. A standard scale backplane with 50 cm reference scale was arranged in the shooting field of view for spatial calibration, with a calibrated spatial measurement accuracy of ±2 mm. This module was used to extract the displacement, attitude and centroid trajectory of the robot during jumping and swimming.

Onboard sensing module: A 9-axis attitude sensor (angle accuracy ±1°, distance accuracy ±2 mm) was mounted at the center of mass of the robot to collect real-time acceleration, angular velocity and attitude angle during locomotion. Four GY-MS5837 pressure sensors (accuracy ±1 kPa) were installed in the gas reservoir and soft combustion chamber to monitor real-time air pressure and provide feedback for the gas filling control system.

Calibration auxiliary module: Standard gauge blocks, electronic balance and gas flowmeter were adopted for pre-experiment calibration. The filling volume of the hydrogen-oxygen mixture (H_2:O_2 = 2:1) was calibrated via the isothermal pressure-volume conversion law before each batch of experiments. The swimming thrust was calculated from onboard acceleration data combined with Newton's second law, and the jumping performance was derived from centroid coordinates extracted frame by frame from high-speed videos.

2. Sampling Frequency and Synchronization Scheme

The sampling frequency of the 9-axis attitude sensor was set to 100 Hz, the GY-MS5837 pressure sensor was set to 200 Hz, and the high-speed camera was set to 240 fps.All devices were triggered synchronously by the main control board: the solenoid valve, ignition device, sensor acquisition and camera recording were activated by the same trigger instruction. In post-processing, the time axes of multi-source data were uniformly calibrated through the characteristic points of take-off impact and stroke start, to ensure the temporal consistency of motion video and sensing data.

3. Number of Trials and Valid Data Screening Criteria

Each working condition was repeated 5 times independently, and valid trials were screened according to the following rules:

For jumping tests: the take-off yaw angle is ≤5° without obvious lateral deviation, the robot does not overturn after landing, the centroid is clearly identifiable throughout the video, and the sensor data has no frame loss or saturation abnormality. The relative standard deviation of jump height/distance of valid samples under the same working condition is ≤8%.

For swimming tests: the robot swims along a straight line with heading deviation ≤10° in a single stroke cycle, and the attitude and pressure data have no abnormal spikes. The relative fluctuation of average swimming speed and peak thrust of valid samples under the same working condition is ≤7%.

Trials that failed to meet the above criteria were excluded. For each experimental group, 3 valid trials with the highest repeatability were selected for final data analysis.

4. Data Post-processing and Statistical Method

Video data processing: The pixel coordinates of the robot's center of mass were extracted frame by frame from high-speed videos, and converted into actual displacement combined with the standard scale backplane, to calculate core performance indicators including jump height, jump distance and swimming speed.

Sensor data processing: A 4th-order Butterworth low-pass filter with a cutoff frequency of 10 Hz was applied to smooth the raw acceleration and pressure data, so as to eliminate environmental vibration and high-frequency noise. The swimming thrust was converted from acceleration integral results combined with the total mass of the robot (613 g).

Statistical method: All performance results are presented as standard deviation. All data calculation, feature extraction and plotting were completed by self-compiled MATLAB programs.

5. Measurement Error and Uncertainty Analysis

The comprehensive measurement uncertainty of this study was synthesized from three main sources:

1. Camera position error caused by pixel resolution and manual calibration, with a value of ±2 mm;
2. Onboard sensor system error, including attitude sensor angle error (±1°) and pressure sensor measurement error (±1 kPa);
3. Gas filling volume error caused by pressure feedback control, with a value of ±0.5 mL.

According to the uncertainty synthesis formula, the relative comprehensive uncertainty of each performance index is calculated as follows: ±4.2% for swimming speed, ±3.8% for jump height, and ±4.5% for jump distance.
