# Supplementary material for: Design, Dynamic Modeling, and Motion Analysis of a Frog-Inspired Hybrid-Driven Amphibious Robot
Source: Sensors (Basel). 2026 Jun 24;26(13):3995. doi: 10.3390/s26133995 (PMC13364168; doi:10.3390/s26133995)
Supplement: Supplementary file 1 [file sensors-26-03995-s001.zip › Files S3 Design of the robot's waterproof seal.pdf]

Design of the robot's waterproof seal

Waterproof sealing is a critical technology for enabling the amphibious robot to perform aquatic movements. It not only protects the stable operation of the robot's various electrical modules but also ensures the safety of the robot while swimming in water. If the waterproof cavity experiences leakage, it may not only damage the electrical components but also lead to a rapid decrease in the robot's buoyancy, thereby disrupting its balance and causing it to capsize and sink. Therefore, it is necessary to implement waterproof sealing designs for the robot system's trunk mechanism, jumping power mechanism, and swimming power mechanism, as shown in figure S6 (a~b). In figure S6 (a), the trunk mechanism is composed of the upper shell, lower shell, and trunk body. Thus, it is necessary to implement sealing treatments between the upper shell and the lower shell, between the upper shell and the trunk body, and between the lower shell and the trunk body. To minimize the sealing treatment required between the lower shell and the trunk body, the lower shell and trunk body have been designed as a single integral component for processing. The remaining two connection points are equipped with sealing grooves B, where waterproof sealant is filled. Additionally, the interface between the trunk body and the explosive chamber cover is also sealed with waterproof sealant in sealing groove A. During assembly, bolts are utilized to achieve mechanical compression and fixation. This design ensures that the connections between the trunk mechanisms form a static seal, thereby achieving waterproof effectiveness.

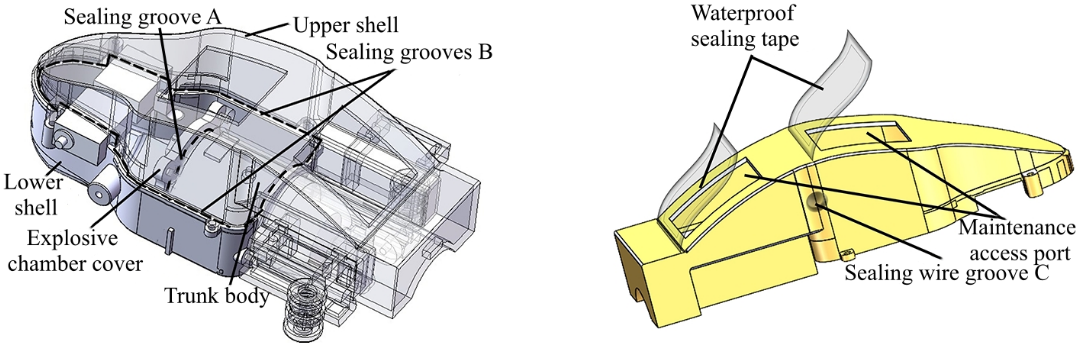

a) Body mechanism seal b) Upper casing seal

Figure S6 Water seal design of robot system

As shown in figure S6 (b), in order to minimize the disruption to the static seals between the trunk mechanisms for inspection and maintenance, two maintenance access ports are incorporated into the upper shell, and waterproof sealing tape is utilized for the static sealing treatment of these access ports. This allows for easy access to inspect the internal drive hardware control modules of the prototype by simply removing the waterproof sealing tape. However, the waterproof sealing tape is only suitable for sealing at shallow water depths, which sufficiently meets the requirements for the experimental research presented in this paper . To ensure stable communication with the servo in the swimming propulsion mechanism, sealing wire groove C is designed on both sides of the shell, allowing the communication lines to be routed into the prototype through this channel. Sealing wire groove C is sealed with waterproof sealant, effectively preventing water ingress. In addition to the aforementioned static sealing treatment, to reduce the overall weight of the robot, waterproof servos are used as the driving source for the actuated joints, enabling direct operation in aquatic environments without the need for complex outer shell structures for dynamic sealing. This design significantly simplifies the mechanical structure while enhancing the stability and reliability of the drive system. Moreover, the use of waterproof servos effectively reduces the risk of sealing failure,

thereby improving the robot's operational capability and long-term reliability in complex underwater environments.
