# Supplementary material for: Design, Dynamic Modeling, and Motion Analysis of a Frog-Inspired Hybrid-Driven Amphibious Robot
Source: Sensors (Basel). 2026 Jun 24;26(13):3995. doi: 10.3390/s26133995 (PMC13364168; doi:10.3390/s26133995)
Supplement: Supplementary file 1 [file sensors-26-03995-s001.zip › Files S4 The Overall Stability of the Robot in Water.pdf]

The Overall Stability of the Robot in Water

1. Criteria for Robot Stability

The stability of the robot's structure is critical for its performance during amphibious movements, particularly when swimming in water. To ensure a smooth and reliable swimming process, the design criteria for the overall structure mandate that the amphibious robot must possess a certain level of stability, which is contingent upon the positions of the center of gravity and the center of buoyancy.When the robot is maneuvering underwater without ascending or descending, the center of buoyancy must be located directly above the center of gravity and aligned with the overall center of the robot. This configuration ensures that the robot remains stable and resistant to capsizing during swimming. The designed jumping and swimming actuation mechanism of the robot achieves a state of self-balancing between the weight and buoyancy during these amphibious movements. Therefore, it is essential to ensure that, in the initial state, the positions of the center of gravity and the center of buoyancy are appropriately aligned, and the magnitudes of the weight and buoyancy are equal.Based on the design and distribution of the components of the prototype described earlier, as shown in figure S7, a coordinate system *O-XYZ* is established with the center of the robot's torso as the origin. G_1 to G_7 represent the gravitational forces acting on the robot's left forelimb mechanism, right forelimb mechanism, top and bottom shell mechanisms, explosive driver, left swimming propulsion mechanism, right swimming propulsion mechanism, and control system hardware. Utilizing the center of mass calculation formula, the position of the robot's center of gravity (X_g, Y_g, Z_g) is determined as follows:

$$X_g = \frac{\sum_{i=1}^{7} \mathbf{M}_X}{G_Z}, Y_g = \frac{\sum_{i=1}^{7} \mathbf{M}_Y}{G_Z}, Z_g = \frac{\sum_{i=1}^{7} \mathbf{M}_Z}{G_Z} \quad \text{(S18)}$$

In the equation, $\mathbf{M}_X$, $\mathbf{M}_Y$, and $\mathbf{M}_Z$ represent the torque due to the gravitational forces of G_1 to G_7 along the *X*, *Y*, and *Z* axes, respectively; G_Z denotes the resultant gravitational force acting on all mechanisms within the system (N).

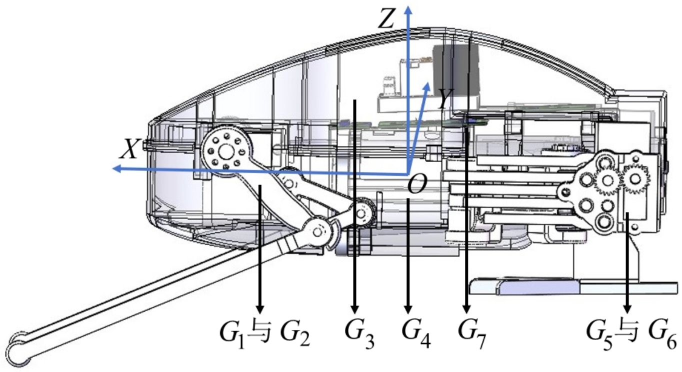

Figure S7 Distribution of robot mechanism centers of mass

Similarly, by incorporating the models of each component within the system, the total drainage volume (Vz) of the robot is determined as follows:

$$V_Z = V_1 + V_2 + V_3 + V_4 + V_5 + V_6 \quad \text{(S19)}$$

In the equation, V_1 and V_2 represent the drainage volumes (ml) of the forelimb mechanisms on the left and right sides, respectively; V_5 and V_6 represent the drainage volumes (ml) of the locomotion power mechanisms on the left and right sides, respectively; V_3 denotes the drainage volume (ml) of the sealed chamber formed by the upper and lower housing mechanisms; V_4 denotes the drainage volume (ml) of the explosive drive unit.

Using equation (S19), the total volume V_z was measured to be approximately 608 ml in the simulation software. By incorporating the total buoyancy of the robot $W_f = \rho g V_z$, the position of the center of buoyancy acting on the robot was determined:

$$X_f = \frac{\sum_{i=1}^{7} \mathbf{W}_X}{W_f}, Y_f = \frac{\sum_{i=1}^{7} \mathbf{W}_Y}{W_f}, Z_f = \frac{\sum_{i=1}^{7} \mathbf{W}_Z}{W_f} \quad \text{(S20)}$$

Where $\mathbf{W}_X$, $\mathbf{W}_Y$, and $\mathbf{W}_Z$ represent the buoyancy moments of G_1 to G_6 along the X, Y, and Z axes, respectively. Based on equations (S20) and (S18), the following criteria for the robot's equilibrium in water are assumed under ideal conditions:

$$\begin{cases} X_g = X_f \\ Y_g = Y_f \\ Z_f \geq Z_g \end{cases} \quad \text{(S21)}$$

According to equation (S21) and in conjunction with the models of various mechanisms within the entire system, if the computed results indicate that $X_g \neq X_f$, $Y_g \neq Y_f$, and $Z_g \neq Z_f$, and the total weight of the system falls within the allowable range, balance can be achieved through appropriate weighting methods. Otherwise, it is necessary to re-optimize the robotic structural design and adjust the positional distribution of the mechanisms.It is known that the overall structure of the robot exhibits a symmetrical distribution on both sides. Therefore, the center of gravity and the center of buoyancy are approximately equal along the Y-axis, while it is challenging to ensure equality along the X and Z axes. To ensure that the robot maintains a certain level of stability in a water environment, the center of gravity and the center of buoyancy should meet the following conditions:

$$\tan\left(\varphi_p\right) = \left| \frac{X_f - X_g}{Z_f - Z_g} \right| \quad \text{(S22)}$$

In the equation, the value of φ_p ranges from 0° to 5°.

2. Robot Stability Verification

As shown in figure S8, first, the actual material properties are assigned to each component of the amphibious robot's three-dimensional model in the simulation software to ensure that it aligns with the real physical environment. Next, the center position of the robot's trunk structure is designated as the origin, allowing the motion coordinate system to maintain consistent alignment with the center position coordinate system, which facilitates subsequent analysis and calculations. Then, the mass properties tool within the software is utilized to calculate the gravitational mass and position coordinates of each module, ensuring accurate mechanical analysis. Similarly, the material properties of the components in the three-dimensional model are uniformly set to those of water. After filling the hollow components, they are saved together with the assembly as a unified model, and the density is set to that of water to ensure that the effects of water are

accurately simulated during the simulation calculations. Subsequently, the mass and position coordinates of the buoyancy are calculated utilizing the software's mass characteristics tool. The detailed information regarding the mass, buoyancy magnitude, as well as the coordinates of the center of mass and center of buoyancy for the robot's structures G_1 to G_7 is provided in Table S1.

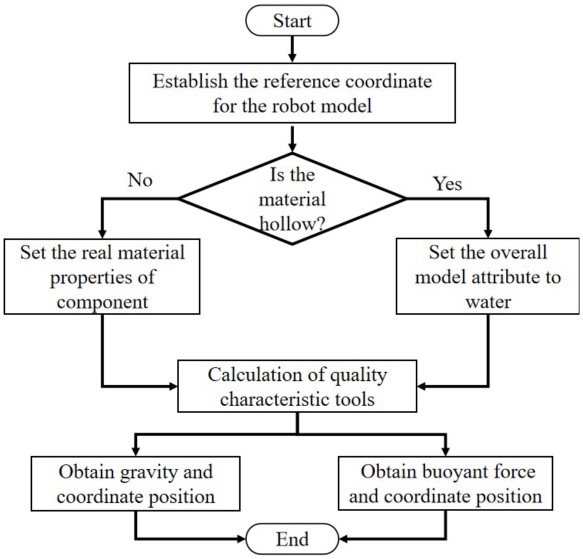

Figure S8 Calculation of buoyancy and gravity of the robot

Table S1 Mass, center of gravity, and center of buoyance of robot components

Serial number G_i	Gravitational mass (g)	Center of mass coordinates (mm)			Buoyant mass (g)	Center of buoyancy coordinates (mm)		
		X	Y	Z		X	Y	Z
1	44	51.29	31.54	8.45	26	61.38	37.94	9.54
2	44	51.29	-31.54	8.45	26	61.38	-37.94	9.54
3	114	15.53	0	-5.67	288	15.45	0.003	6.94
4	185	-33.92	-0.01	-18.18	194	-29.92	-0.01	-10.18
5	71	-19.29	66.43	7.60	44	-19.46	69.31	9.73
6	71	-19.29	-66.43	7.60	44	-19.46	-69.31	9.73
7	82	35.44	0.01	30.92	-	-	-	-

The serial numbers in table S1 represent the left front limb mechanism, the right front limb mechanism, the upper and lower shell mechanisms, the explosive driver, the left swimming power mechanism, the right swimming power mechanism, and the hardware of the control system. By substituting the data from the table into equations (S18) and (S20), the magnitude and position coordinates of the center of mass and buoyant force are obtained, as shown in table S2.

Table S2 Overall gravity, buoyancy and coordinates of the robot

Category	Center of gravity point			Buoyancy center		
Force（g）	611			622		
Coordinate	X_g	Y_g	Z_g	X_f	Y_f	Z_f
(mm)	1.7564	0	3.4856	1.244	0	13.7612

From Table S2, it is indicated that the overall center of mass force of the robot virtual prototype is 10 g less than the buoyant force. Therefore, balance can be achieved by adding a few grams of weight to the

prototype model Based on the coordinate values in the table, it can be determined that Z_f - Z_g = 10.2756 mm, with $Z_f > Z_g > 0$, $Y_g = Y_f = 0$, $|X_f - X_g| = 0.5124$ mm. Utilizing these calculated values and applying formula (S22), we can derive that $\varphi_p \approx 2.8$ degrees, which satisfies the balance criterion. This confirms that the overall structure of the designed frog-like amphibious robot exhibits good stability in water, ensuring a steady posture during its swimming motion.
